# Mitochondrial translation is the primary determinant of secondary mitochondrial complex I deficiencies

**DOI:** 10.1016/j.isci.2024.110560

**Published:** 2024-07-19

**Authors:** Kristýna Čunátová, Marek Vrbacký, Guillermo Puertas-Frias, Lukáš Alán, Marie Vanišová, María José Saucedo-Rodríguez, Josef Houštěk, Erika Fernández-Vizarra, Jiří Neužil, Alena Pecinová, Petr Pecina, Tomáš Mráček

**Affiliations:** 1 Laboratory of Bioenergetics, Institute of Physiology, Czech Academy of Sciences, 14200 Prague, Czech Republic; 2 Department of Biomedical Sciences, University of Padova, 35131 Padova, Italy; 3 Veneto Institute of Molecular Medicine, 35129 Padova, Italy; 4 Department of Genetics and Microbiology, Faculty of Science, Charles University, 12800 Prague, Czech Republic; 5 Laboratory for Study of Mitochondrial Disorders, First Faculty of Medicine, Charles University and General University Hospital, 12808 Prague, Czech Republic; 6 Department of Cell Biology, Faculty of Science, Charles University, 12800 Prague, Czech Republic; 7 School of Pharmacy and Medical Science, Griffith University, Southport, Qld 4222, Australia; 8 Laboratory of Molecular Therapy, Institute of Biotechnology, Czech Academy of Sciences, 25250 Prague, Czech Republic; 9 Department of Pediatrics and Inherited Diseases, First Faculty of Medicine, Charles University, 12108 Prague, Czech Republic; 10 Department of Physiology, Faculty of Science, Charles University, 12800 Prague, Czech Republic

**Keywords:** Biochemistry, Molecular biology, Cell biology

## Abstract

Individual complexes of the mitochondrial oxidative phosphorylation system (OXPHOS) are not linked solely by their function; they also share dependencies at the maintenance/assembly level, where one complex depends on the presence of a different individual complex. Despite the relevance of this “interdependence” behavior for mitochondrial diseases, its true nature remains elusive. To understand the mechanism that can explain this phenomenon, we examined the consequences of the aberration of different OXPHOS complexes in human cells. We demonstrate here that the complete disruption of each of the OXPHOS complexes resulted in a decrease in the complex I (cI) level and that the major reason for this is linked to the downregulation of mitochondrial ribosomal proteins. We conclude that the secondary cI defect is due to mitochondrial protein synthesis attenuation, while the responsible signaling pathways could differ based on the origin of the OXPHOS defect.

## Highlights

• Loss of either OXPHOS complex leads to secondary complex I downregulation
• Complex I level correlates with the content of mitochondrial ribosome subunits
• Secondary complex I deficiency is caused by mitochondrial translation attenuation
• Signaling pathways are dysregulated differently among individual OXPHOS defects

## Graphical abstract

**Figure:**
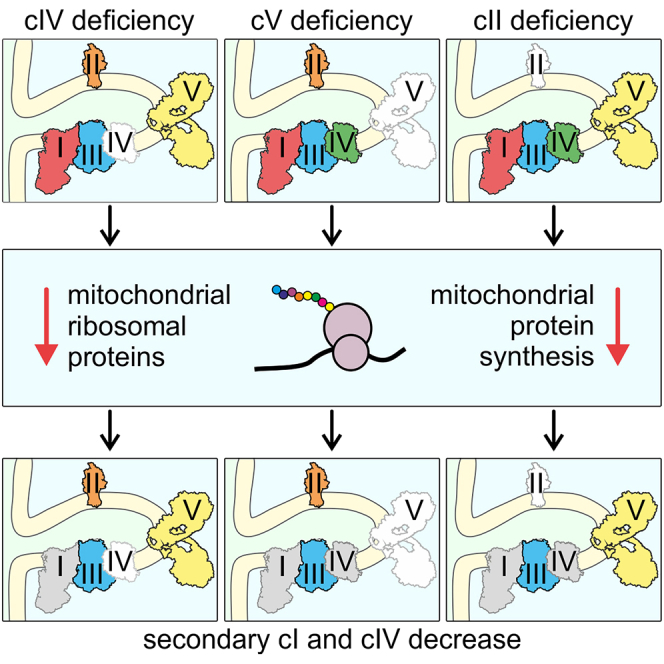

## Introduction

Biogenesis of individual multimeric enzymes of the mitochondrial oxidative phosphorylation system (OXPHOS), consisting of the respiratory chain (RC) complexes I (NADH dehydrogenase, cI), II (succinate dehydrogenase, cII), III (cytochrome *bc*_*1*_ complex, cIII), and IV (cytochrome *c* oxidase, cIV), plus the F_1_F_o_-ATPase (cV), is a highly complex process due to the dual genetic origin of individual complex subunits. Even though the vast majority of mitochondrial proteins are encoded by nuclear DNA (nDNA), 13 OXPHOS structural subunits (7 of cI, 1 of cIII, 3 of cIV, and 2 of cV subunits) are coded for by mitochondrial DNA (mtDNA).^1^^,^^2^ Expression of these subunits relies on autonomous replication, transcription, and translation systems within mitochondria, including the presence of structurally and functionally distinct mitochondrial ribosomes.^3^^,^^4^^,^^5^ Therefore, the expression of individual subunits of mitochondrial complexes in the cytosol and mitochondria needs to be strictly coordinated to avoid proteostatic stress.^6^^,^^7^

An additional level of complexity is given by the interaction of cI, cIII, and cIV, forming structures known as respiratory supercomplexes (SCs).^8^ In mammalian mitochondria, the structures of the respirasome (i.e., the SC I III_2_IV),^9^^,^^10^^,^^11^ SC I III_2_,^12^ SC III_2_IV^13^ and the megacomplex (SC I_2_III_2_IV_2_)^14^ have been recently resolved by cryo-EM. However, the exact functional significance of respiratory SCs is still unclear.^15^ Among several roles proposed for the SCs are the possible improved efficiency of electron transfer by means of channeling, protection against ROS production, or involvement in the assembly and/or stabilization of individual complexes.^16^^,^^17^ The last mentioned role for the SCs was proposed to explain RC complex interdependence, i.e., the observation that defects in one complex affect the level and function of other complexes.^18^^,^^19^

RC complex interdependence may help to explain the biochemical features observed in some patients with mitochondrial disease.^20^ For example, individuals carrying mutations affecting MT-CYB, the mtDNA-encoded subunit of cIII, present biochemically as a cI and cIII combined deficiency.^21^^,^^22^^,^^23^^,^^24^^,^^25^ In addition, severe defects in cIV biogenesis are associated with a secondary cI defect.^26^^,^^27^^,^^28^^,^^29^^,^^30^ Conversely, the absence of MT-CO2, which blocks cIV assembly in later stages, enables the stabilization of cI associated with cIII_2_ and with partially assembled cIV submodule containing MT-CO1, other structural subunits, and the assembly factor HIGD2A.^31^ A secondary decrease in cI level was proposed to stem from its active degradation due to the oxidative damage triggered by highly reduced coenzyme Q pool, causing reverse electron transfer (RET) and the ensuing oxidative stress.^29^ However, different explanations for this phenomenon have been proposed, including a block in cI maturation in human cybrid cells lacking MT-CYB^25^ or a shutdown of mitochondrial translation in cells with a profound early assembly cIV defect due to the absence of the COX4 subunit.^30^ Moreover, it has been proposed that cIV biogenesis is regulated by the interplay between the translation of MT-CO1 and its assembly via the mitochondrial translation regulation assembly intermediate of cytochrome *c* oxidase (MITRAC) complexes.^32^^,^^33^ One of the MITRAC components, COA1 or MITRAC15, has also been described as a cI assembly factor necessary for the correct translation of MT-ND2,^34^^,^^35^^,^^36^ possibly linking cI and cIV biogenesis.

Specific signaling pathways developed in mammals to mediate the inter-organelle crosstalk under stress due to the various types of mitochondrial dysfunctions. For instance, the energetic deprivation of cells combined with a higher level of ROS has been reported to promote the activation of the AMP-dependent kinase (AMPK), which inhibits the mechanistic target of rapamycin complex 1 (mTORC1), a major regulator of cytosolic protein synthesis,^37^^,^^38^ including nuclear-encoded mitochondrial components.^39^ Moreover, recent studies described the role of the mitochondrial stress sensing OMA1-DELE1-HRI pathway in the initiation of integrated stress response (ISR) in mammals.^40^^,^^41^ Mitochondrial ISR had been shown to alter the nuclear expression program as a response to mitochondrial dysfunction induced by various stimuli.^42^^,^^43^^,^^44^^,^^45^^,^^46^^,^^47^ Therefore, AMPK-mTORC1 axis and ISR represent possible signaling pathways to underlie OXPHOS complex interdependence.

Here, we have systematically analyzed the phenomenon of cI interdependence using a collection of human KO cell lines deficient for various components of OXPHOS, in particular structural subunits of cIV (COX4I1 and COX6B1) and cV (ATP5F1B). Our results have shown that a profound cI deficiency is associated with a shutdown in mitochondrial translation. To better understand the adaptive responses leading to decreased mitochondrial translation, we have explored the possible involvement of several regulatory pathways, such as the AMPK-mTORC axis^39^ and the ISR.^40^^,^^41^^,^^42^^,^^45^^,^^46^ In summary, we present compelling data that helps our understanding of how modifications to OXPHOS composition adapt mitochondrial physiology to different OXPHOS deficiencies.

## Results

### Severe oxidative phosphorylation system deficiencies cause a secondary decline of the other oxidative phosphorylation system components, especially of complex I

**Figure 1:**
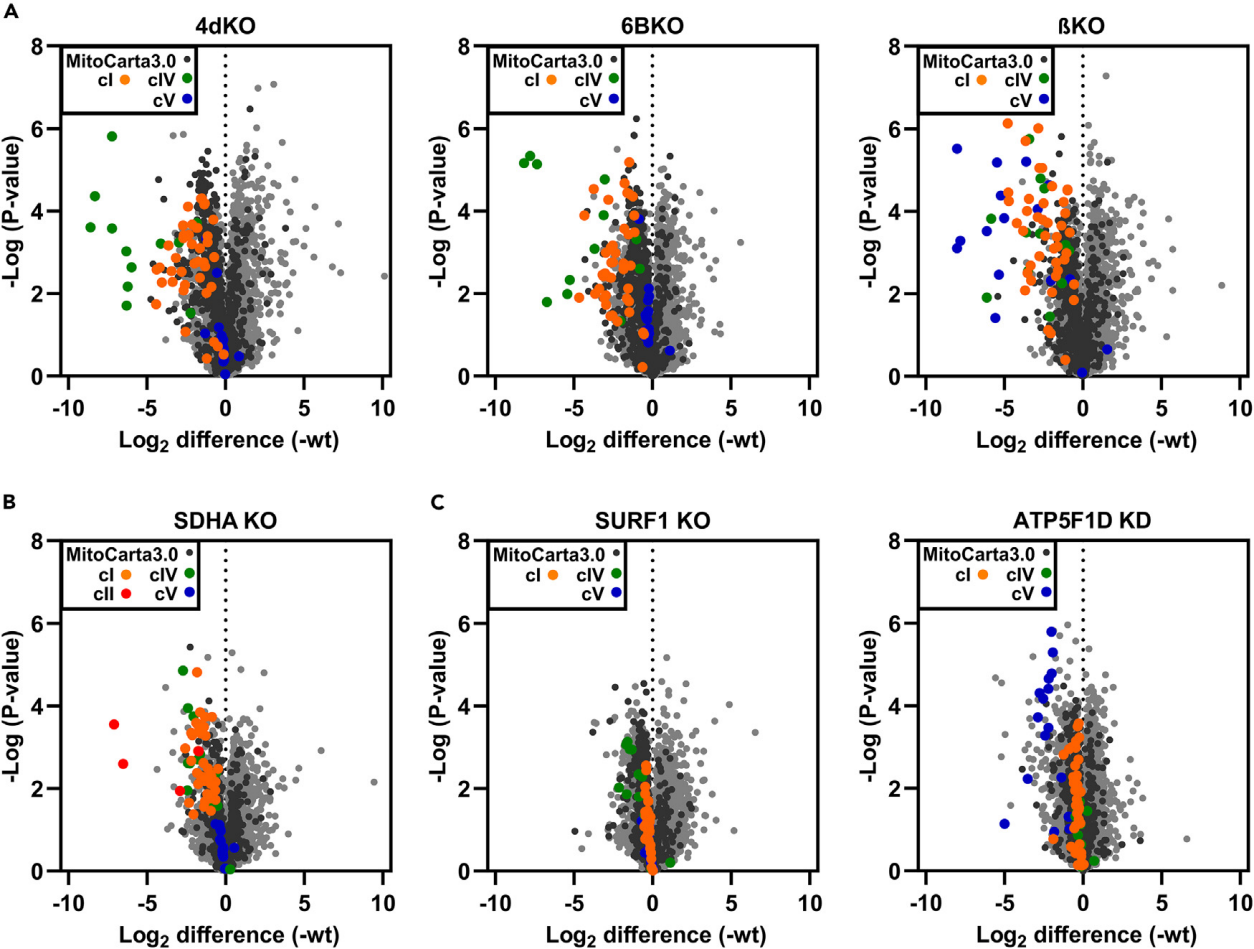
Severe OXPHOS deficiencies cause secondary decline of the other OXPHOS components, especially of cI (A) Differential content of mitochondrial proteins between wt and 4dKO, 6BKO (cIV), and βKO (cV) cells. (B) Differential content of mitochondrial proteins between wt and SDHA KO cells (cII; See also Figure S1). (C) Differential content of mitochondrial proteins between wt and SURF1 KO (cIV) or ATP5F1D KD (cV) cells (See also Figure S1). Volcano plots represent LFQ-MS analysis (*n* = 3) of all analyzed proteins (gray), MitoCarta3.0 annotated proteins (black), and subunits of cI (orange), cII (red), cIV (green) and cV (blue).

Deficiency of HEK293 cells in the cytochrome *c* oxidase (cIV) early assembly structural subunit COX4 (4dKO) leads to a profound secondary cI defect.^30^^,^^48^ To better understand the phenomenon of OXPHOS complex interdependence, we expanded our analyses using additional KO cell lines with severe OXPHOS deficiency of different origin. Our major focus was on two of them, COX6B1 (cIV) and ATP5F1B (cV) knock-out cells. We chose cells with a deficiency of COX6B1 (6BKO cells), since its absence also causes severe cIV deficiency, yet contrary to 4dKO, it affects the late steps of cIV assembly and preserves the initial assembly modules formed by subunits MT-CO1, COX4, and COX5A.^49^ The ATP5F1B knock-out cell line (termed βKO) lacking the beta subunit of cV^50^ served as an RC-independent model of OXPHOS deficiency. The relative abundance of mitochondrial proteins in each OXPHOS-deficient model was determined by label-free quantification mass spectrometry (LFQ-MS). Similar to the observations in 4dKO cells (cIV),^30^ lower levels of cIV and cV subunits in cIV- (6BKO) and cV-(βKO) deficient cells, respectively, were accompanied by a profound decrease in the level of cI subunits. In addition, βKO cells (cV) also showed a secondary decrease in the abundance of cIV subunits (Figure 1A).

Since we observed a profound secondary decrease of cI subunit levels in severe cIV (4dKO, 6BKO) and cV (βKO) deficiencies, we asked whether this is a general phenomenon. Therefore, we examined HEK293 cells deficient in the cII catalytic subunit SDHA (SDHA KO), which showed a secondary decrease in cI and cIV levels (Figures 1B and S1). Additionally, a secondary decrease in cI and cIV subunit protein levels is also associated with severe cIII deficiency.^25^^,^^51^ Thus, current and past evidence shows that severe defects in one component of the OXPHOS system trigger a secondary effect on cI and cIV. However, we also demonstrated that this phenomenon can only be observed when the OXPHOS defect is severe, as cell lines with milder deficiencies of either cIV (SURF1 KO) or cV (ATP5F1D KD) did not show the interdependence effect (Figures 1C and S1).

### Alternative oxidase restores mitochondrial respiration in cIV- but not in cV-deficient cells

A previous report suggested that the secondary cI decrease in OXPHOS-deficient models could be caused by a redox imbalance.^29^ Introduction of alternative oxidase (AOX) with CoQ:O_2_ oxidoreductase activity^52^ in mouse and human cells can bypass RC components downstream of CoQ and normalize redox balance.^53^ Indeed, the alleviation of the reduced CoQ by AOX expression was previously shown to increase cI levels in cells with profound cIII and cIV defects.^25^^,^^29^^,^^54^ To test whether AOX recovers cI levels in our models, we stably introduced AOX (HA-tagged, from *Aspergillus nidulans*)^55^ in cIV- (4dKO, 6BKO) and cV- (βKO) deficient cells (Figures S2A and S2B).

**Figure 2:**
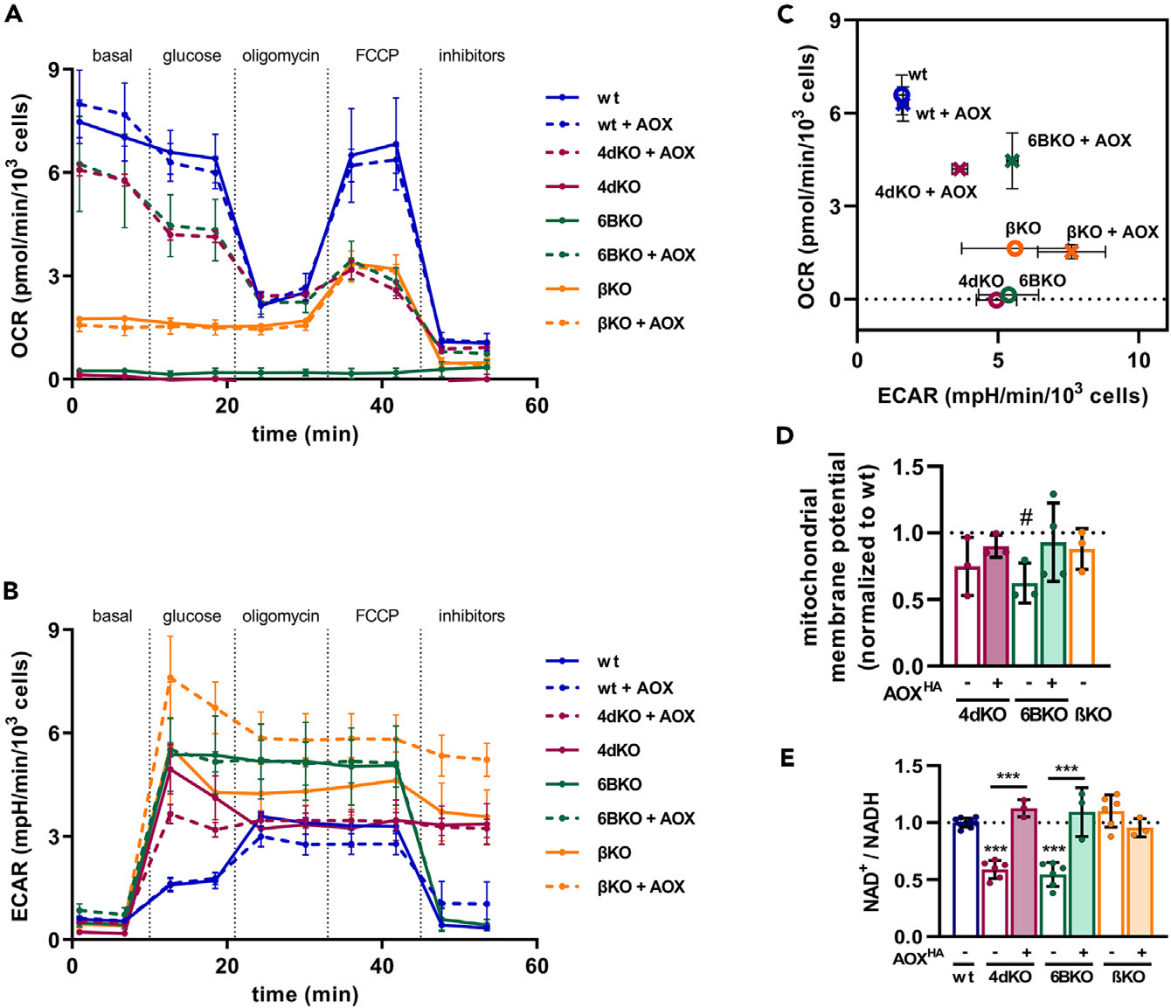
Alternative oxidase expression restores mitochondrial respiration in cIV- but not in cV-deficient cells (A) Evaluation of cellular oxygen consumption rate (OCR) and (B) extracellular acidification rate (ECAR) in the wt, KO models (full lines) and the cell lines expressing AOX (dashed lines) normalized to cell number. In the case of 4dKO ± AOX and βKO ± AOX cell lines, the 2-deoxyglucose addition (inhibitors - a mixture of rotenone, antimycin A, 2-deoxyglucose and Hoechst 33342) led to cells detaching and therefore was omitted from the measurement (*n* ≥ 3, mean ± SEM). (C) Metabolic phenotype of wt, 4dKO, 6BKO, and βKO cells (circle), and 4dKO AOX, 6BKO AOX, and βKO AOX cells (cross) represented as a phenogram combining parallel evaluation of cellular oxygen consumption rate (OCR) and extracellular acidification rate (ECAR) (*n* ≥ 3, mean ± SEM). (D) Mitochondrial membrane potential relative to the wt (mean ± SD). See also Figure S2C. (E) NAD+/NADH ratio assessed in the cellular models (mean ± SD). One sample t-test (#*p* < 0.05), or One-way ANOVA (∗*p* < 0.05; ∗∗*p* < 0.01; ∗∗∗*p* < 0.001) was performed (*n* ≥ 3). wt, 4dKO, 6BKO (cIV) and βKO (cV) ± AOX cell lines were utilized.

Evaluation of the “bioenergetic phenotype,” i.e., the cellular oxygen consumption rate (OCR) vs. extracellular acidification rate (ECAR), showed null OCR in cIV-deficient cells (4dKO, 6BKO) and significantly decreased OCR in βKO (cV) cells (Figures 2A and 2C). ECAR in the OXPHOS-deficient cells (4dKO, 6BKO, βKO) indicated a higher glycolytic rate (Figures 2B and 2C), providing ATP by substrate-level phosphorylation. AOX expression in cIV-deficient cells (4dKO, 6BKO) significantly increased OCR, indicating a partial recovery of electron flux through the RC (Figures 2A and 2C). However, AOX expression did not reduce the high ECAR observed in cIV-deficient models (4dKO, 6BKO; Figures 2B and 2C). Contrarily, AOX expression in cV-deficient cells (βKO) did not produce any change in the “bioenergetic phenotype” (Figure 2C).

We then focused on additional bioenergetic parameters related to OXPHOS (dys)function. Cells with the three severe OXPHOS deficiencies (4dKO, 6BKO, and βKO) maintained mitochondrial membrane potential (Figures 2D and S2C). In 6BKO (cIV) cells, mitochondrial membrane potential was slightly reduced, yet this reduction was alleviated by AOX expression (Figures 2D and S2C). On the contrary, the mitochondrial membrane potential in βKO (cV) cells did not differ from wt cells, which is surprising, given that the absence of ATP synthase would in principle prevent the dissipation of the proton gradient. We also determined the NAD^+^/NADH ratio, which was decreased in cIV-deficient cells (4dKO, 6BKO) but was restored to wt levels by AOX expression (Figure 2E). In cV-deficient cells (βKO), the NAD^+^/NADH ratio was comparable to that of wt cells and remained unchanged upon AOX expression (Figure 2E). This may reflect the notion that these cells preserve some cI function (Figures 2A and 2C), but it also indicates that cV-deficient cells (βKO) are not confronted with NADH reductive stress, one of the possible triggers of the secondary cI decrease.

### Alternative oxidase expression restores complex I levels and cristae ultrastructure in cytochrome *c* oxidase-deficient cells

**Figure 3:**
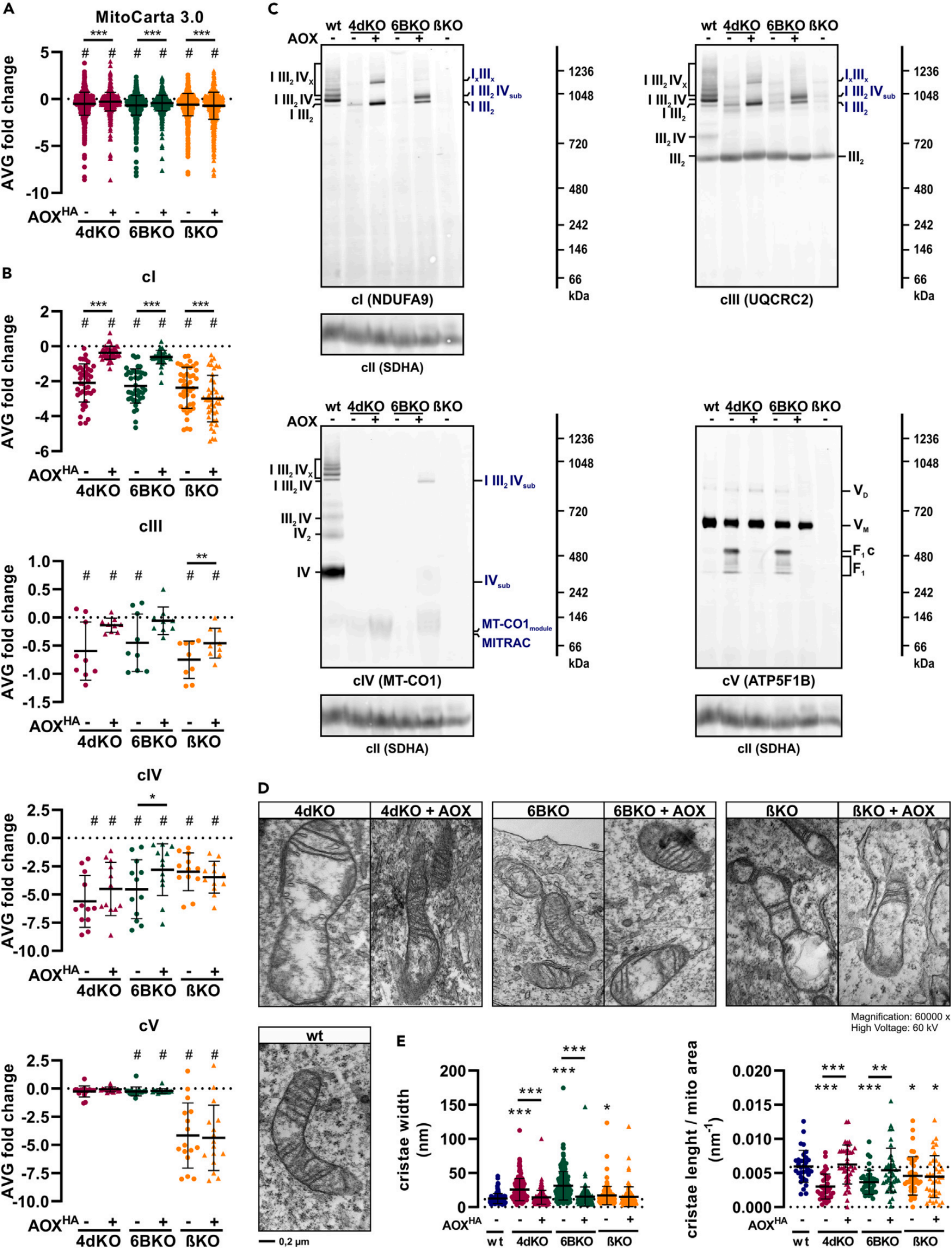
AOX expression restores cI levels and cristae ultrastructure in cIV-deficient cells (A) LFQ-MS-based average fold-change of MitoCarta3.0 annotated proteins relative to wt (*n* = 3). (B) LFQ-MS-based average fold-change of individual subunits of OXPHOS complexes (cI, cIII, cIV, cV) relative to wt (*n* = 3). See also Figure S3. (C) Blue-native (BN)-PAGE/WB detection of OXPHOS complexes: cI (NDUFA9 antibody), cIII (UQCRC2 antibody), cIV (MT-CO1 antibody), and cV (ATP5F1B antibody). Antibody against cII (SDHA) was used as a loading control (*n* = 2). NDUFA9 and UQCRC2 antibodies were consecutively developed in two different fluorescent channels, and have the same (SDHA) loading control. (D) Representative transmission electron microscopy (TEM) images of mitochondria in OXPHOS-deficient models. (E) TEM-based quantification of cristae length per mitochondrial area and cristae width. One sample t-test (#*p* < 0.05), or One-way ANOVA (∗*p* < 0.05; ∗∗*p* < 0.01; ∗∗∗*p* < 0.001) was performed. Data are presented as mean ± SD. wt, 4dKO, 6BKO (cIV) and βKO (cV) ± AOX cell lines were utilized.

**Figure 4:**
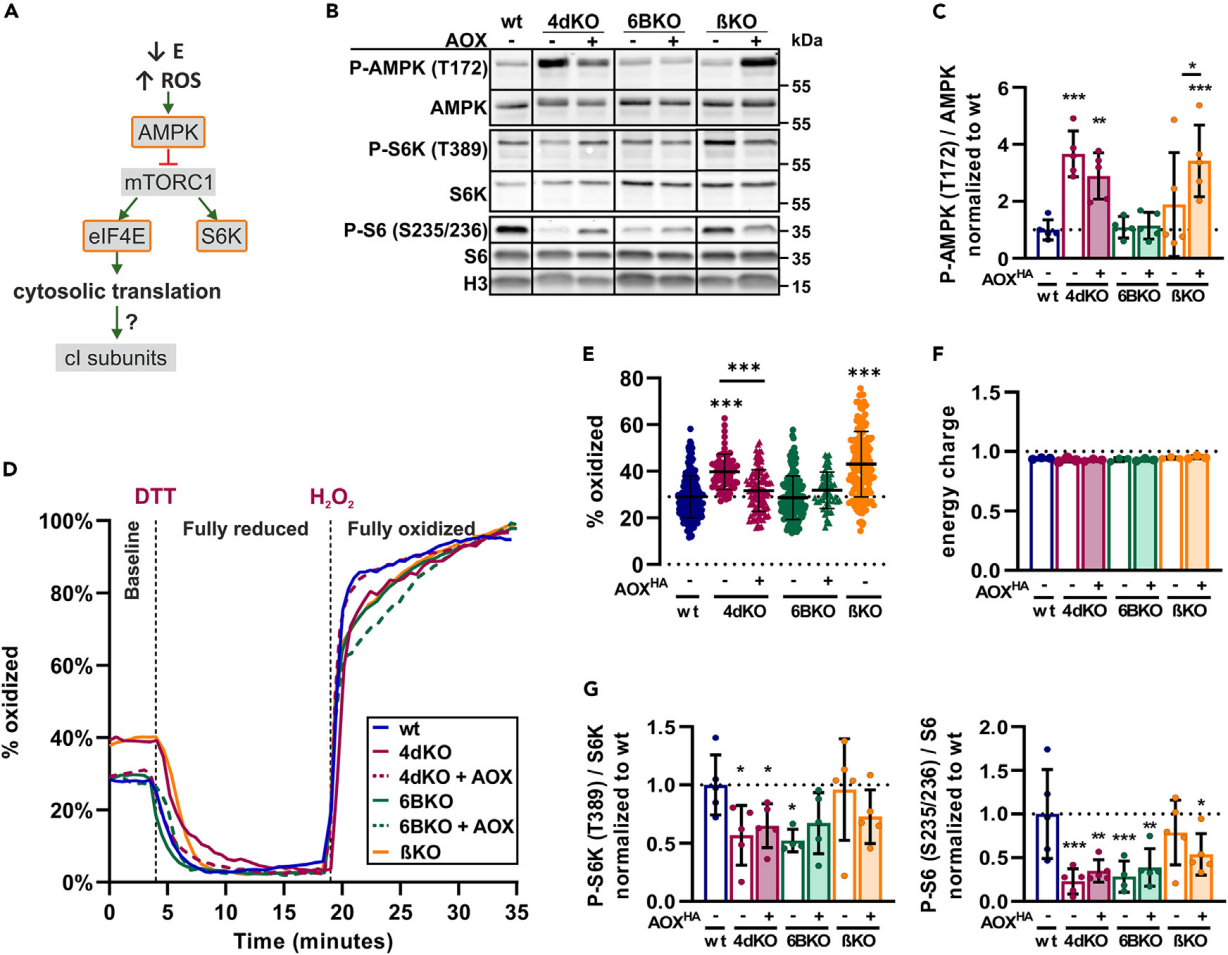
The AMPK-mTORC1 axis is not consistently activated in all the OXPHOS-deficient models (A) Scheme of AMPK-mTORC1 axis signaling, with highlighted examined parts (orange edge). (B) Representative SDS-PAGE/WB analysis of the AMPK (T172), S6K1 (T389), and S6 ribosomal protein (S235/236) phosphorylation, and corresponding loading control (H3) in whole cell lysates. (C) Quantification of phosphorylated to total AMPK ratio normalized to the wt (*n* = 3). (D) Representative traces of mito-roGFP measurements. Each line represents the average of normalized values from one experiment (*n* ≥ 18 cells). The average baseline value of each cell was considered as one data point for the final result in Figure 4E. (E) Confocal microscopy-based analysis of mitochondrial redox status using mito-roGFP, expressed as a percentage of the maximal oxidation of the sensor. Each dot represents an individual cell (*n* ≥ 55). (F) Energy charge of the studied cells (*n* = 3). The energy charge was calculated using the formula: ([ATP]+[ADP]/2)/([ATP]+[ADP]+[AMP]). See also Figure S4B. (G) Quantification of phosphorylated to total S6K1 or S6 ratio normalized to the wt. Histone H3 (H3) antibody was used as a loading control (*n* = 3). One-way ANOVA (∗*p* < 0.05; ∗∗*p* < 0.01; ∗∗∗*p* < 0.001) was performed. Data are presented as mean ± SD. wt, 4dKO, 6BKO (cIV) and βKO (cV) ± AOX cell lines were utilized.

**Figure 5:**
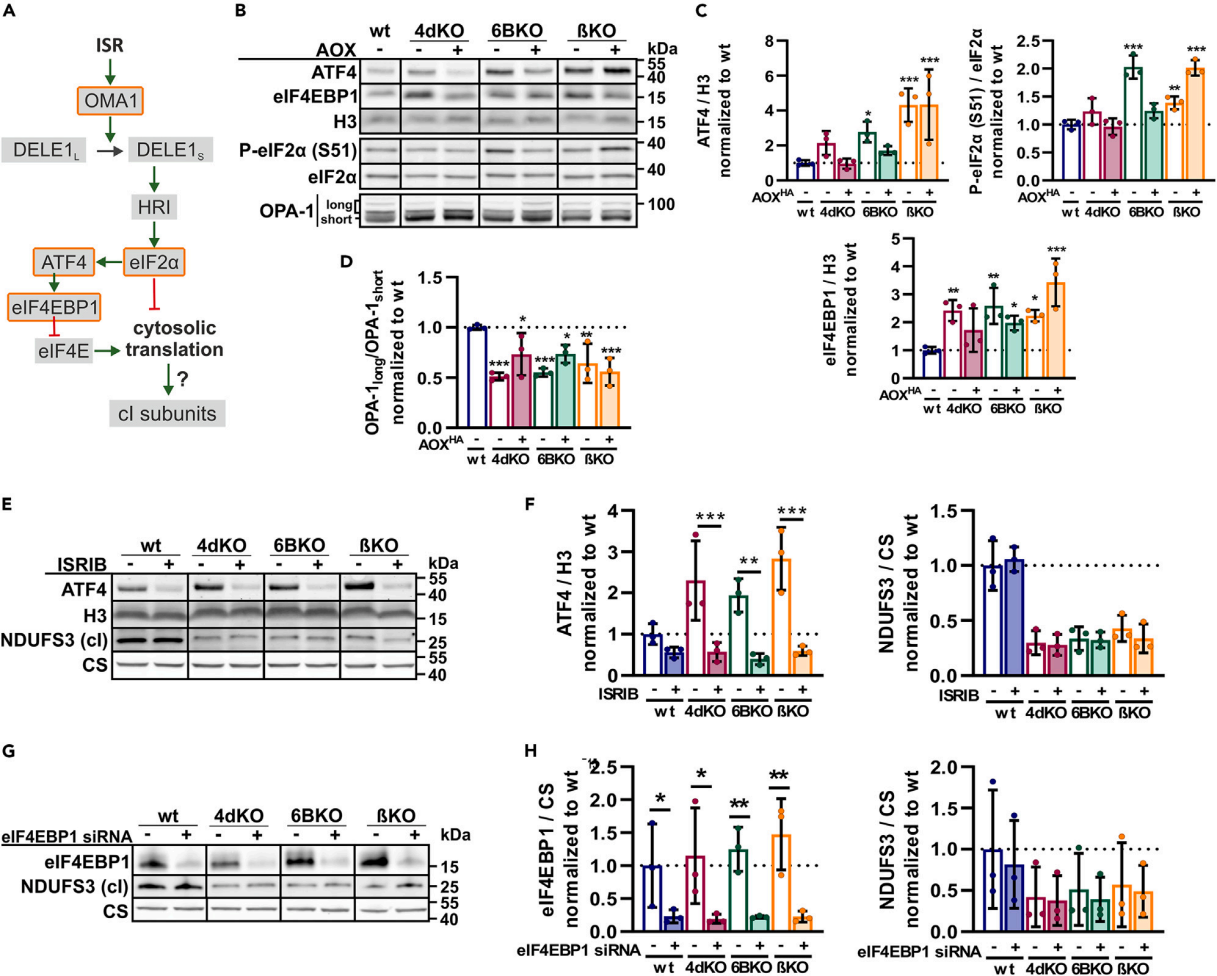
Primary cIV and cV deficiency activates the mitochondrial integrated stress response that is counteracted by AOX expression in cIV-deficient cells (A) Scheme of OMA1-DELE1-HRI signaling toward integrated stress response (ISR), with highlighted examined parts (orange edge). (B) Representative SDS-PAGE/WB analysis of the protein steady-state levels of ATF4 and eIF4EBP1, eIF2α phosphorylation (S51), and long (OPA-1_long_) and short (OPA-1_short_) OPA-1 in whole cell-lysates. (C) Quantification of phosphorylated (S51) to total eIF2α ratio normalized to the wt, and quantification of ATF4 and eIF4EBP1 signals normalized to H3. (D) Quantification of OPA-1_long_ to OPA-1_short_ ratio. (E) Representative SDS-PAGE/WB analysis of the protein steady-state level of ATF4 and NDUFS3 (cI), and H3 and CS as the loading controls, in whole cell lysates of the cells with DMSO addition (−), and after 48 h incubation with 1uM ISRIB (+). See also Figure S5. (F) Quantification of the ATF4 signal normalized to H3, and NDUFS3 signal normalized to CS. (G) Representative SDS-PAGE/WB analysis of the protein steady-state levels of eIF4EBP1 and NDUFS3 (cI) in whole cell lysates of the cells transfected with scrambled siRNA (−), or transfected with eIF4EBP1 siRNA (+). See also Figure S5. (H) Quantification of eIF4EBP1 signal normalized to H3, and NDUFS3 signal normalized to CS. One-way ANOVA (∗*p* < 0.05; ∗∗*p* < 0.01; ∗∗∗*p* < 0.001) was performed (*n* = 3). Data are presented as mean ± SD. wt, 4dKO, 6BKO (cIV) and βKO (cV) ± AOX cell lines were utilized.

**Figure 6:**
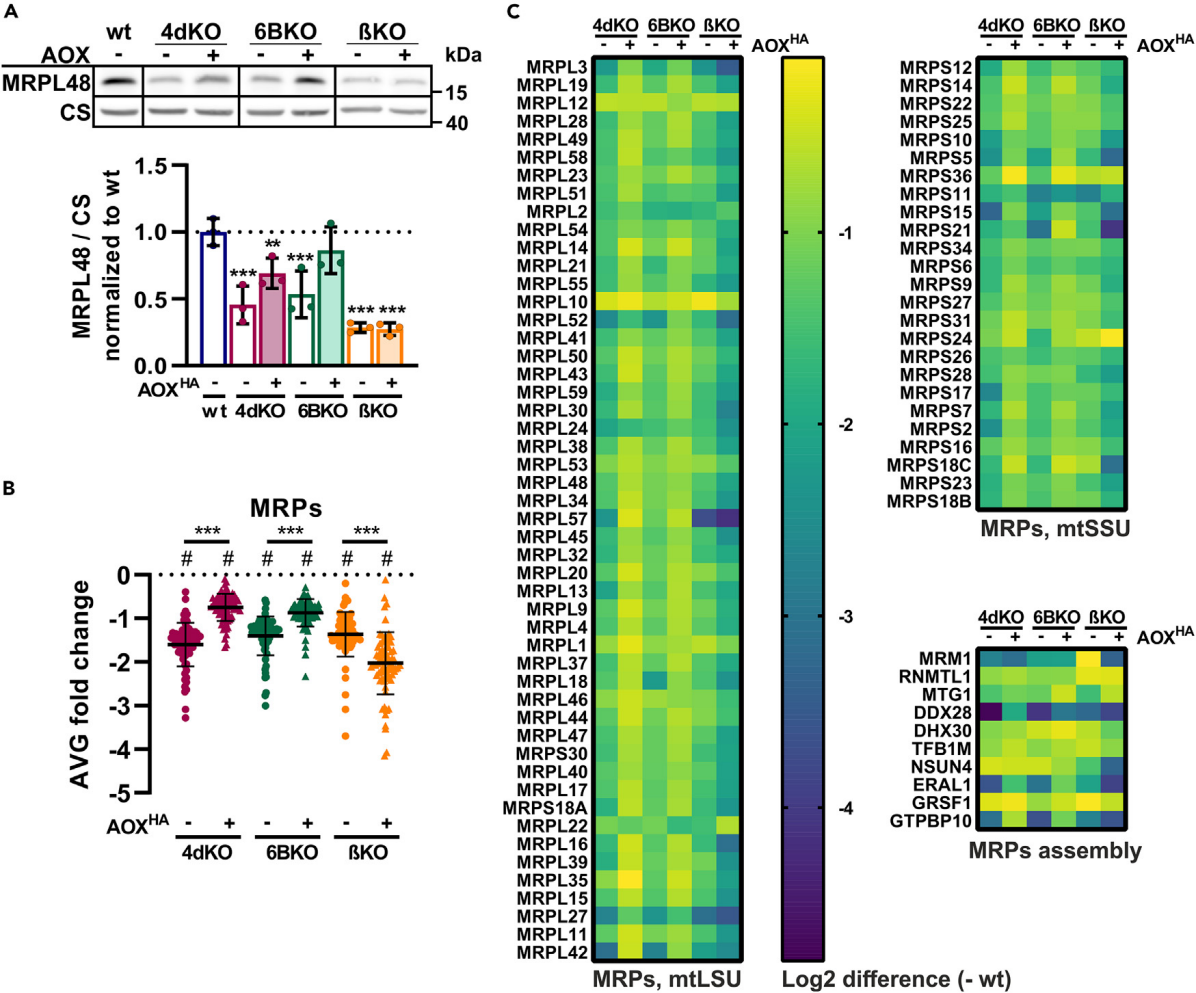
Mitochondrial ribosomal protein levels are decreased in severe OXPHOS-deficient cells (A) Representative SDS-PAGE/WB analysis of the steady-state levels of MRPL48 in whole cell-lysates. CS antibody was used as a loading control. Graph represents the quantification of the MRPL48 signal normalized to CS. (B) LFQ-MS-based average fold-change of mitochondrial ribosomal proteins (MRPs) relative to wt. (C) Heat-maps depict the LFQ-MS-based average fold-change relative to wt of MRPs forming large subunit of mitochondrial ribosome (mtLSU), small subunit (mtSSU), and assembly factors of the mitochondrial ribosome (MRPs assembly). See also Figure S5G. One sample t-test (#*p* < 0.05), or One-way ANOVA (∗*p* < 0.05; ∗∗*p* < 0.01; ∗∗∗*p* < 0.001) was performed (*n* = 3). Data are presented as mean ± SD. wt, 4dKO, 6BKO (cIV) and βKO (cV) ± AOX cell lines were utilized.

**Figure 7:**
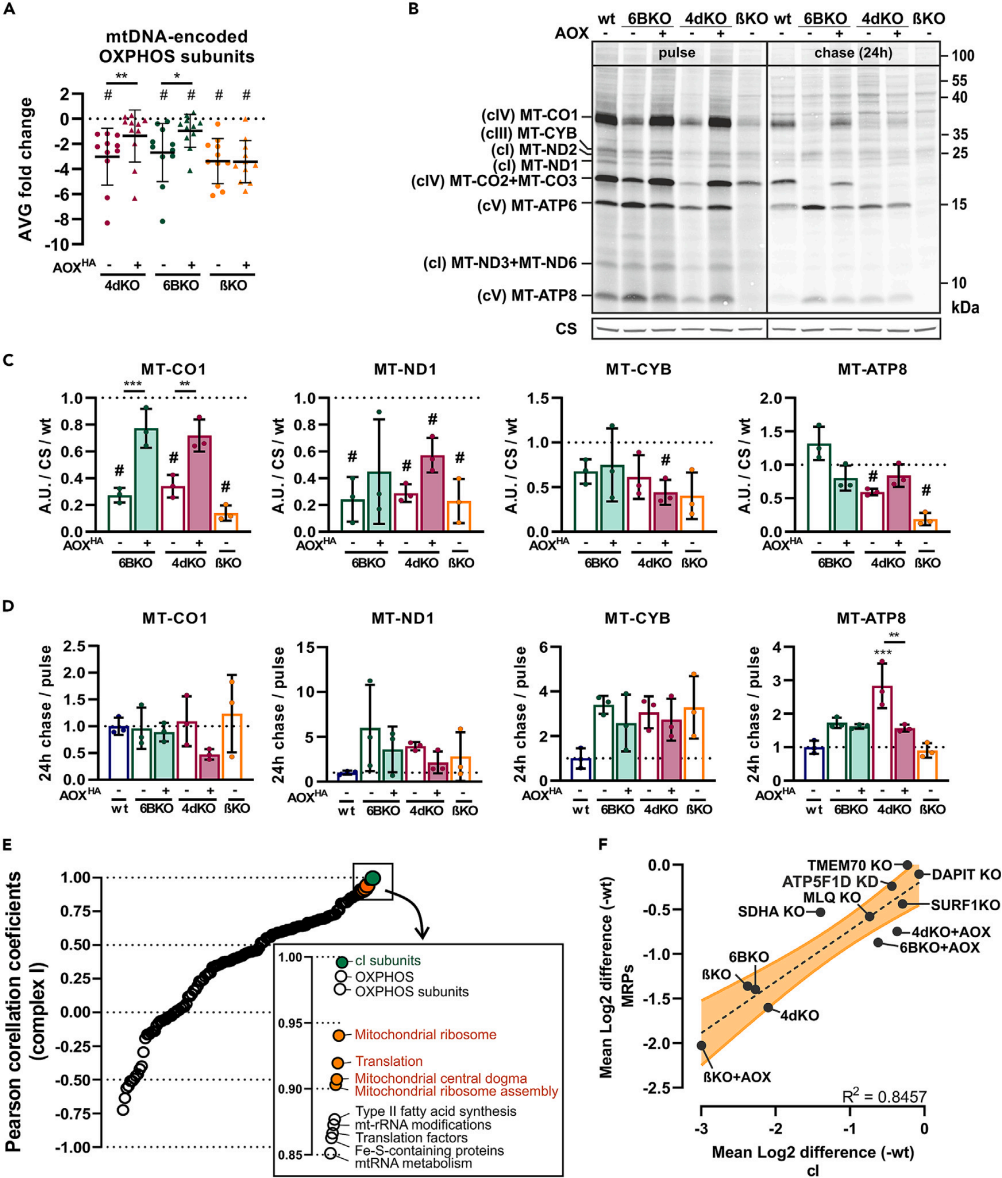
Mitochondrial translation attenuation reflects the severity of OXPHOS deficiency (A) LFQ-MS-based average fold-changes of mtDNA-encoded proteins (*n* = 3). (B) Representative autoradiographic detection of ^35^S *in vivo* labeled mtDNA-encoded OXPHOS subunits in whole cell lysates analyzed by SDS-PAGE. See also Figure S6. (C) Quantification of pulse ^35^S *in vivo* labeling signal of representative cIV (MT-CO1), cI (MT-ND1), cIII (MT-CYB), and cV (MT-ATP8) subunits normalized to CS, and relative to wt (*n* = 3). (D) Quantification of 24 h chase to pulse ratio of ^35^S *in vivo* labeling signal of representative cIV (MT-CO1), cI (MT-ND1), cIII (MT-CYB), and cV (MT-ATP8) subunits (*n* = 3). (E) Pearson correlation coefficients of LFQ-MS based average fold-changes comparison of complex I with MitoCarta3.0 annotated categories relative to wt in HEK293-cell line-based models of i) severe deficiency: SDHA KO (cII, *n* = 3), 4dKO (cIV, *n* = 3), 6BKO (cIV, *n* = 3), βKO (cV, *n* = 3), and ii) mild deficiency: SURF1 KO (cIV, *n* = 3), ATP5F1D KD (cV, *n* = 2), MLQ KO (cV, *n* = 2), DAPIT KO (cV, *n* = 3), TMEM70 KO (cV, *n* = 3). See also Figures S6C and S6D. (F) Linear regression comparing LFQ-MS based average fold-changes of MRPs and cI subunits relative to wt in HEK293-cell line-based models of i) severe deficiency: SDHA KO (cII, *n* = 3), 4dKO (cIV, *n* = 3), 6BKO (cIV, *n* = 3), βKO (cV, *n* = 3), and ii) mild deficiency: SURF1 KO (cIV, *n* = 3), ATP5F1D KD (cV, *n* = 2), MLQ KO (cV, *n* = 2), DAPIT KO (cV, *n* = 3), TMEM70 KO (cV, *n* = 3). One sample t-test (#*p* < 0.05), or one-way ANOVA (∗*p* < 0.05; ∗∗*p* < 0.01; ∗∗∗*p* < 0.001) was performed (*n* = 3). Data are presented as mean ± SD. wt, 4dKO, 6BKO (cIV) and βKO (cV) ± AOX cell lines were utilized.

Our LFQ-MS data indicate that in the three KO cell lines (4dKO, 6BKO, and βKO), there was a slight, yet significant, decrease in the overall abundance of mitochondrial proteins (based on MitoCarta3.0 database),^56^ which was counteracted by AOX expression in the two cIV-deficient cell types (4dKO, 6BKO; Figure 3A). A separate analysis of the steady-state levels of the subunits of each of the OXPHOS complexes, both using SDS-PAGE/WB and immunodetection with specific antibodies (Figures S3A and S3B) and by means of LFQ-MS analysis (Figures 3B, S3C, and S3D), revealed that the abundance of cI and cIV subunits was substantially lower than the average of the mitochondrial proteome in the three cell lines (4dKO, 6BKO, βKO; Figures 3B and S3). In addition, βKO (cV) cells showed the downregulation of all cV subunits (Figure 3B), while cIV-deficient models (4dKO, 6BKO) presented only a mild decrease of cV mtDNA-encoded subunits MT-ATP6, MT-ATP8 (Figure S3D). Even though the level of the UQCRC2 subunit of cIII was significantly decreased in the three OXPHOS deficiency models (4dKO, 6BKO, βKO; Figure 1, Figure 2, Figure 3, Figure 4, Figure 5, Figure 6, Figure 7S3A and S3B), levels of cIII and cII subunits were not overall reduced to the same extent as levels of cI subunits (Figures 3B, S3C, and S3D). Interestingly, the levels of cI subunits were recovered by AOX expression in cIV-deficient cells (4dKO, 6BKO) but remained low or were even further reduced in cV-aberrant cells (βKO; Figures 3B and S3).

Given the observation of low steady-state levels of OXPHOS proteins in the KO cell models (4dKO, 6BKO, and βKO), we next tested the assembly state of OXPHOS complexes and SCs by BN-PAGE/WB. Consistently, the level of cI in all its native forms was much lower in 4dKO (cIV) cells, as shown previously.^30^ In 6BKO (cIV) cells, cI was only present within the I III_2_ SC, probably due to the loss of fully assembled cIV, associated with the accumulation of MT-CO1 in low molecular weight subcomplexes (Figure 3C). In accordance with the low steady-state levels of cI and cIV subunits observed previously, all fully assembled forms of cI and cIV were also severely lower in βKO (cV) cells (Figure 3C). AOX expression in cIV-deficient cells (4dKO, 6BKO) resulted in a pronounced increase in the level of cI in SCs. Consistent with the proteomics data (Figure 3B), the total level of fully assembled cIII was only mildly decreased and was mainly present in the dimeric free form (III_2_) in 4dKO, 6BKO (cIV), and βKO (cV) cells. After AOX expression in cIV-lacking cells (4dKO, 6BKO), cIII was associated with above-mentioned SCs (Figure 3C). Levels of the cV monomer and dimer were not changed in cIV-deficient cells (4dKO, 6BKO). However, the cV F_1_ domain sub-assemblies that were detected in cIV KO cells disappeared after AOX expression (Figure 3C). To ensure that changes in cI content and assembly status depend on the AOX function and not just its structural presence, we also analyzed the effect of AOX expression in wt cells. Here, neither the content of individual subunits for cI, cIV or cV nor the pattern of assembled cI forms changed in comparison to wt cells (Figures S3E and S3F).

Stability and assembly of SCs are modulated by cristae morphology^57^ and, vice versa, mitochondrial cristae morphology is affected by OXPHOS function, as documented for cIV^58^^,^^59^ or cV defects.^60^ To determine the effect of OXPHOS deficiency on mitochondrial cristae formation in our cell lines, we performed transmission electron microscopy (TEM). All OXPHOS deficiency models (4dKO, 6BKO, and βKO) showed perturbed mitochondrial morphology (Figure 3D), with cristae being shorter and wider than in wt cells (Figure 3E). These cristae defects were significantly improved by AOX expression in cIV-deficient cells (4dKO, 6BKO), consistent with the observed partial restoration of RC electron flux and RC assembly. On the contrary, AOX expression did not influence cristae ultrastructure in the cV-deficient model (βKO; Figures 3D and 3E).

In summary, severe OXPHOS aberrations in HEK293 cells lead to a specific decrease in cI. AOX expression causes a reversal to the wt phenotype in cIV-, but not in cV-deficient cells.

### The AMP-dependent kinase-mechanistic target of rapamycin complex 1 axis is not consistently activated in all oxidative phosphorylation system-deficient models

To determine signaling pathways that may trigger cI attenuation in our KO models (4dKO, 6BKO, βKO), we examined the involvement of the AMPK-mTORC1 axis, which could be activated by a drop in mitochondrial ATP production and increased ROS generation^37^^,^^38^^,^^39^ (Figure 4A). AMPK was activated by phosphorylation,^61^ which was evident in 4dKO (cIV) cells, where the level of phosphorylated T172 of AMPK (P-AMPK) was significantly higher than in wt cells, and was reduced after AOX expression (Figures 4B and 4C). In contrast, AMPK phosphorylation was observed neither in 6BKO (cIV) nor in βKO (cV) cells. Interestingly, AOX expression induced AMPK activation in βKO (cV) cells, suggesting that AOX may be detrimental to the energetic status of βKO cells (Figures 4B and 4C). We asked whether the disparities in AMPK activation were due to differences in mitochondrial ROS production or the energy charge, i.e., the ratio between the different phosphorylation states of adenine nucleotides (AMP, ADP, and ATP) among the different cell models. 4dKO (cIV) and βKO (cV) cells produced significantly more ROS than wt cells, while there was no increase in ROS production in 6BKO (cIV) cells (Figures 4D, 4E, and S4A). AOX expression reduced ROS production in 4dKO (cIV) cells, but in βKO (cV) cells, the effect was the opposite, increasing ROS formation (Figures 4D, 4E, and S4A). Since in 4dKO AOX (cIV) cells, ROS production decreased to levels comparable to wt cells, yet AMPK remained highly phosphorylated, and since βKO (cV) cells showed increased ROS without an increase in P-AMPK levels, we conclude that AMPK phosphorylation in these cells is ROS-independent. The differences in P-AMPK could not be explained by a drop in ATP levels relative to ADP or AMP, as energy charge remained the same in all cell lines (Figures 4F and S4B).

AMPK activation inhibits the mTORC1 pathway, a major regulator of cytosolic translation.^37^ We therefore tested whether this pathway is dysregulated in our cell models (4dKO, 6BKO, and βKO) and whether mTORC1 inactivation (either via AMPK-dependent or independent pathways) could explain the observed general decline in cI subunit levels (Figure 4A). We analyzed the phosphorylation status of S6K1, the main mTORC1 phosphorylation target, which is activated by phosphorylation at T389 and then phosphorylates the ribosomal protein S6. S6K1 phosphorylation was significantly reduced in 4dKO and 6BKO cells (cIV; Figures 4B and 4G). AOX expression increased S6K1 phosphorylation only in 6BKO (cIV) cells, and it remained unchanged in βKO and βKO AOX (cV) cells (Figures 4B and 4G). Phosphorylation of S6 (S235/236) was significantly decreased in our cIV deficiency models (4dKO, 6BKO), but not in βKO cells (cV; Figures 4B and 4G). The decrease in the phosphorylation of mTORC1 targets in OXPHOS-deficient cells could provide a possible explanation of the decrease in cI if it originated from a decrease in the cytosolic translation of nuclear-encoded cI components. However, the expression of AOX did not result in the activation of these targets, ruling out mTORC1 inhibition as the cause of the secondary cI decline. Overall, AMPK-mTORC1 signaling was dysregulated across the spectrum of the studied OXPHOS-deficient models (4dKO, 6BKO, βKO). But since alterations in the AMPK-mTORC1 axis differed between models, they cannot act as a general “mediator” of cI downregulation.

### Primary cIV and cV aberrations activate the mitochondrial ISR, which is counteracted by AOX expression in cIV-deficient cells

We then tested the possible role of the ISR (ISR, Figure 5A), which has been associated with OXPHOS dysfunction,^45^^,^^46^ in the observed secondary cI downregulation. For this purpose, we analyzed the phosphorylation status of eIF2α (S51) and the level of ATF4 and eIF4EBP1 in cIV- (4dKO, 6BKO) and cV- (βKO) lacking cells (Figures 5B and 5C). Compared to wt cells, the ATF4 and eIF4EBP1 steady-state levels as well as eIF2α phosphorylation were significantly increased in cIV- (4dKO, 6BKO) and cV- (βKO) deficient models but were normalized by AOX expression only in cIV-deficient cells (4dKO, 6BKO) (Figures 5B and 5C). Mitochondrial ISR is known to activate the OMA1 protease (Figure 5A), which then cleaves the mitochondrial dynamin-like GTPase OPA1.^40^^,^^41^^,^^62^ The three KO cell lines (4dKO, 6BKO, and βKO) showed a significantly decreased long-to-short OPA1 ratio, indicating a higher OPA1 cleavage rate (Figures 5B and 5D). AOX expression partially elevated this ratio in cIV-deficient cells (4dKO, 6BKO), while it remained unchanged in βKO (cV) cells. Accordingly, mitochondria were significantly more fragmented in OXPHOS-deficient cells, with no changes in mitochondrial mass (Figure S5C), and this fragmentation was reverted by AOX expression only in cIV-deficient cells (4dKO, 6BKO; Figures S5A–S5C). Taken together, the disruption of OXPHOS function by severe deficiencies of either cIV or cV results in the activation of mitochondrial ISR, and in cIV-deficient cells, it can be reversed by the restoration of the electron flux in the RC by AOX expression. To unequivocally determine whether mitochondrial ISR is the reason for OXPHOS complex interdependence, we suppressed mitochondrial ISR activation using two different approaches: (i) incubation of cells with an ISR inhibitor (ISRIB), and (ii) silencing eIF4EBP1 expression. Despite efficient inhibition of ISR in cIV- (4dKO, 6BKO) and cV- (βKO) lacking cells, documented by (i) a significant decrease of ATF4 and its targets to wt levels (Figures 5E and 5F) and (ii) decreased eIF4EBP1 levels (Figures 5G and 5H), the level of cI subunits was not increased (Figures 5E–5H and S5D–S5F).

Thus, both cIV and cV deficiency trigger mitochondrial ISR signaling, presumably via the OMA1-DELE1-HRI pathway. While the chronic activation of ISR may be relevant for cellular adaptation to severe OXPHOS deficiency, it is not involved in the OXPHOS complex interdependence phenomenon.

### Mitochondrial ribosomal protein levels are decreased in severe models of cIV and cV deficiency

Since we concluded that neither the AMPK-mTORC1 nor the ISR signaling pathways are responsible for the secondary effect on cI in our KO cells (4dKO, 6BKO, βKO), we searched for other possible reasons. LFQ-MS data analysis of mitochondrial proteins revealed that mitochondrial ribosomal proteins (MRPs), the constituents of both large (mtLSU) and small (mtSSU) mitochondrial ribosome subunits, were in general two to four times decreased in cIV- (4dKO, 6BKO) and cV- (βKO) deficient cells compared to wt (Figures 6A–6C). Interestingly, not only the structural components were decreased, but this was also the case for proteins involved in the assembly of the mitochondrial ribosome (Figure 6C). Nevertheless, this decrease was more homogeneous in the case of MRPs, while the most affected assembly factors of mitochondrial ribosomes were DDX28, ERAL1, and GTPBP10. Similar to cI subunits (Figure 3B), the expression of AOX increased the level of MRPs in cIV-deficient cells (4dKO, 6BKO) but not in βKO (cV) cells (Figures 6B and 6C). This phenocopies the AOX effect on the restoration of electron flux in cIV-deficient cells, since AOX expression in wt cells did not influence MRP levels either (Figure S5G).

### Attenuation of mitochondrial translation reflects the severity of oxidative phosphorylation system aberration

mtDNA-encoded polypeptides were among the most severely reduced proteins in our OXPHOS-deficient models (4dKO, 6BKO, βKO; Figure 7A). To determine whether this decrease resulted from the attenuation of mitochondrial protein synthesis and/or faster degradation, we performed pulse-chase metabolic labeling (Figure 7B). The synthesis of the mtDNA-encoded cIV and cI subunits was the most affected in cIV- (4dKO, 6BKO) and cV- (βKO) deficient cells, which was reduced to 30% of wt (Figures 7B, 7C, and S6A). MT-ATP8, and MT-ATP6 protein levels after the pulse were reduced in cV-deficient cells (βKO), while their translation was mostly unchanged in cIV-KO (4dKO, 6BKO) cells. In contrast, mtDNA-encoded subunit of cIII (MT-CYB) was only mildly decreased in our KO models (4dKO, 6BKO, βKO), without a sign of significance. Expression of AOX increased the newly synthesized mtDNA-encoded subunits of cIV and cI in cIV-deficient cells (4dKO, 6BKO; Figures 7B, 7C, and S6A). Interestingly, the recovery of MT-CO2+MT-CO3 synthesis after AOX expression was much more noticeable in 6BKO cells than in 4dKO cells (Figures 7B and S6A). This may be related to the stabilization of the MT-CO2 subunit within the subcomplexes and within SCs we observed using BN-PAGE/WB analysis (Figure 3C).

Detection of the remaining labeled proteins following a 24-h chase in the non-labeling medium allowed the estimation of turnover of mtDNA-encoded proteins (Figures 7B, 7D, and S6B). In general, the protein levels decreased in all analyzed cell lines, including wt. Interestingly, cIV subunits did not show a significant change in the chase-to-pulse ratio compared to wt cells, indicating similar turnover rates. On the other hand, cI and cIII subunits were even more stable in all OXPHOS-deficient models than in wt cells.

Altogether, these results indicate that the reason behind the secondary decline in the steady-state levels of cI and cIV mtDNA-encoded subunits is the attenuation of mitochondrial translation, most probably related to the observed decrease in the abundance of the mitochondrial ribosome components. To investigate this link, we analyzed our LFQ-MS data for correlation between cI subunits and other MitoCarta3.0-annotated categories across the spectrum of OXPHOS deficiency models with varying degree of secondary cI downregulation. This included the 6BKO, 4dKO (cIV), βKO (cV), and SHDA (cII) models used in this work, but also a spectrum of less severe models, i.e., SURF1 KO (cIV), ATP5F1D KD,^63^ MLQ KO,^64^ DAPIT KO, and TMEM70 KO^50^ (cV) cells. We calculated the matrix of Pearson correlation coefficients of LFQ-MS-based Log2 abundance changes across individual annotation terms (Figure S6C), from which it is apparent that cI terms show strong correlation with mitochondrial protein synthesis terms. Indeed, cI subunits have the strongest correlation with mitochondrial translation components (Figure 7E), and this is true also for the opposite correlation of mitochondrial ribosome components to cI subunits (Figure S6D). As a proof of principle, cV subunits do not present such a strong correlation with mitochondrial translation components, based on the same dataset (Figure S6E). These data imply a strong cross-dependence between cI and mitochondrial ribosome abundance (Figures 7E, S6C, and S6D). We also plotted LFQ-MS-based Log2 abundance changes of cI subunits (x axis) and MRPs (y axis), from which it is apparent that the extent of cI subunit decrease correlates with diminishing content of MRPs (84,57% accuracy and *p* value < 0.0001) (Figure 7F).

## Discussion

The cross-dependencies in the assembly and/or stability of individual RC complexes (with cI being the most frequent secondary “target”) have often been documented, yet the underlying molecular mechanism(s) are not fully understood. Secondary cI decline was first observed in patients with mitochondrial disease with severe cIII deficiency caused either by mutations in the mtDNA cIII gene *MT-CYB* or in factors involved in its synthesis and assembly.^21^^,^^23^^,^^25^^,^^65^^,^^66^ Subsequently, this phenomenon was observed in a number of mouse and human cell models with primary severe cIII and cIV aberrations,^26^^,^^27^^,^^28^^,^^29^ including our previously characterized 4dKO HEK cell model.^30^

We have now significantly deepened the knowledge about the “interdependence phenomenon” by showing that the level of cI is also decreased in additional cell models with severe RC aberrations of cII (SDHA KO) and cIV (6BKO), as well as in a KO model of cV (βKO), which, although being a member of the OXPHOS system, it is not an RC complex. The decrease in the steady-state level of cI subunits was not surprising in 4dKO cells, in which the cIV deficiency is due to the disruption of cIV assembly at very early stages.^30^ The reason being that the molecular defect would be similar to that of Cox10 KO mouse cells^26^ or human MT-CO1 mutated cybrids,^27^^,^^31^ in which the interdependence phenomenon had also been observed. However, we also observed a profound functional and structural cIV defect in 6BKO cells, which was surprising because the COX6B1 subunit is thought to be incorporated within the final steps of cIV assembly,^1^ and it does not impair the formation of early assembly precursors containing MT-CO1. This would indicate that it is not necessarily the disruption of the initial steps of cIV assembly that causes the secondary cI defect, but rather the severity of the OXPHOS aberrations caused by the primary defect. In fact, the observation of interdependence of cI and cIV with ATP synthase in cells devoid of the beta subunit (βKO),^50^ and cII in cells lacking SDHB^67^ or SDHA, proves that lack of cI maintenance or assembly is due to a severe OXPHOS dysfunction and not because of impaired respirasome formation, as both cII and cV are absent from SCs.^68^ Moreover, different degrees of OXPHOS impairment have a different outcome as the interdependency phenotype was not observed in cell lines with milder OXPHOS aberrations of either cIV (SURF1 KO) or cV (ATP5F1D KD). We have now documented that the interdependence manifestation strongly depends on the severity of the primary defect and that it is not limited to RC deficiencies but rather represents a general adaptive mechanism to the impairment of oxidative phosphorylation.

Even though the first reports describing the interdependence phenomenon could not discern whether the secondary cI decline in mouse cells with severe defects of cIII and cIV was due to an assembly defect or possible destabilization of cI, some reports proposed that it was likely due to impaired cI assembly.^23^^,^^26^^,^^27^ However, a more detailed study of relevant mechanisms suggests that the reason is the active degradation of fully assembled cI triggered by excessive ROS production originated by a high ratio of reduced to oxidized CoQ, favoring RET. The reason behind this interpretation is either the maintenance of cells under hypoxia or expression of the AOX resulting in the stabilization of cI.^29^ Nonetheless, this is not what was observed when studying human cybrids with a very severe cIII defect due to homoplasmic 4-bp deletion in *MT-CYB*. In these cells, the detailed analysis of cI assembly kinetics showed that there was a block in the maturation of the complex.^25^ In addition, these cells also displayed a cIV defect, which was explained by an assembly defect and by the accumulation of partially assembled cIV intermediates. Expression of AOX in the MT-CYB Δ4-bp cybrids partially recovered cI activity, pointing to a role of the redox state in cI biogenesis.^18^^,^^25^ Further, ROS production was increased in 4dKO but not in 6BKO cells (cIV), which displayed a similarly profound reduction in cI levels as the former cell model. Therefore, increased ROS production is not directly responsible for cI decline. Even though we cannot rule out the possibility of oxidative damage of cI in some of our cell models, this will probably not translate into severely decreased cI subunit levels. In a recent study describing the phenotype of DNAJC30 mutations compromising the turnover of oxidative damage-prone subunits of the cI N-module, the levels of cI subunits remained unchanged, and only its activity decreased.^69^ Further, SDHB KO (cII) in HEK293 cells showed cI downregulation that occurred within the time frame of weeks. Apparently, in the case of SDHB KO, this served as an adaptation to normalize the mitochondrial NAD^+^/NADH ratio to allow for a sufficient rate of aspartate synthesis required for cell proliferation.^67^ Such a progressive adaptation process is likely independent of acute changes in oxidative stress but would rather be governed by a mechanism involving decreased biogenesis of cI.

Our previous analyses of 4dKO (cIV) cells suggested that the downregulation of cI in the absence of cIV originates in the early biogenesis stages, i.e., at the level of mitochondrial translation.^30^ We have now significantly expanded our understanding of the molecular causes of the interdependence phenomenon, in which the attenuation of mitochondrial translation was observed in all different cell lines with severely dysfunctional OXPHOS. However, fully assembled cIII and cV were only marginally affected in cIV-deficient cells (4dKO, 6BKO). The presence of combined defects of cI and cIV is common in patients with mitochondrial disease with defects in mitochondrial translation^70^ and may be due to higher stability of the cIII and cV components.^71^^,^^72^ Since we now show that the interdependence phenomenon is principally governed by impaired mitochondrial protein synthesis, it implicates that cI and cIV are most prone to secondary downregulation. We have unequivocally established this for cI, while the βKO (cV) and SDHA KO (cII) models, where the primary defect is not associated with cIV deficiency, display not only secondary cI but also secondary cIV downregulation.

As it was observed in other models,^25^^,^^29^ AOX expression results in a recovery of cI levels and respiratory rates also in our cIV-deficient models (4dKO, 6BKO). Interestingly, this was accompanied by a significant elevation of the level of MRPs and an increased rate of mtDNA-encoded protein translation. In contrast, expressing AOX in βKO (cV) cells rescued neither MRPs nor cI or cIV deficiencies. The difference is most likely because the OXPHOS defect in these cells is not at the level of the RC. Nevertheless, we hypothesized that the introduction of AOX would still decrease the H^+^/e^−^ ratio and therefore increase the electron transfer rate from NADH to oxygen even under conditions of strict respiratory control associated with the lack of cV. However, this was not the case as respiration rates and NAD^+^/NADH ratios remained unchanged after the expression of AOX in βKO (cV) cells.

It is still not clear what triggers the decrease in MRPs. First, we evaluated the mitochondrial membrane potential, the decrease of which could explain a possible low rate of MRPs reaching the mitochondrial matrix, as membrane potential is essential for the import of many proteins from the cytosol to mitochondria.^73^^,^^74^ However, all OXPHOS-deficient models (4dKO, 6BKO, βKO) maintained a significant membrane potential. Next, we hypothesized that this was related to signaling pathways sensing OXPHOS dysfunction. We then explored the AMPK-mTORC1 axis and mitophagy driven by energetic deprivation,^37^^,^^38^ which did not provide a plausible explanation either. The ISR^40^^,^^41^ originating in mitochondria is emerging as a signaling pathway involved in metabolic adaptations under various stress conditions.^42^^,^^43^^,^^44^^,^^45^^,^^46^^,^^47^ The ISR was consistently activated in all our models (4dKO, 6BKO, βKO), and the response was alleviated by AOX expression in the two cIV-lacking cell lines (4dKO, 6BKO), but not in cV-KO (βKO). However, inhibiting the ISR both pharmacologically and genetically did not recover MRPs (Figures S5E and S5F) or cI levels. It is possible that OXPHOS dysfunction-sensing mechanisms differ across the models, yet they do converge at the downregulation of mitochondrial ribosomes and decreased rate of mitochondrial protein synthesis. Therefore, the molecular mechanisms that underlie the interdependence phenomenon seem to be linked to different redox-mediated pathways responding to the stress generated by OXPHOS dysfunction in general, warranting further investigation.

### Limitations of the study

This study demonstrates that the downregulation of mitochondrial protein synthesis is the primary trigger for secondary cI downregulation in models of OXPHOS deficiencies, while the exact sensing/signaling mechanism is yet to be fully uncovered. Our cellular models demonstrate that the main factors determining this phenomenon are neither respirasome-based structural interactions nor degradation of oxidatively damaged holo-complex I. However, with our current data, we cannot fully discard the possibility that ROS could still play a secondary, albeit relevant role. For this, full-scale redox proteomics of complex I subunits would be required to ultimately determine whether this is the case. Also, our work has been performed only in a single human cell line. Since interdependency is limited to severe OXPHOS defects, tissue-specific knockouts would present plausible animal models. However, dynamics of the assembly of OXPHOS complexes seem to differ between human and rodent models^75^; therefore, human cell lines represent a model relevant for patients with mitochondrial diseases, where the phenomenon of interdependence has been observed.

## STAR★Methods

### Key resources table

**Table:** 

REAGENT or RESOURCE	SOURCE	IDENTIFIER
**Antibodies**		

NDUFA9 antibody	Abcam	Cat# ab14713, RRID:AB_301431
NDUFB8 antibody	Abcam	Cat# ab110242, RRID:AB_10859122
NDUFS3 antibody	Abcam	Cat# ab110246, RRID:AB_10861972
SDHA antibody	Abcam	Cat# ab14715, RRID:AB_301433
UQCRC2 antibody	Abcam	Cat# ab14745, RRID:AB_2213640
UQCRC2 antibody	Proteintech	Cat# 14742-1-AP, RRID:AB_2241442
MTCO1 antibody	Abcam	Cat# ab14705, RRID:AB_2084810
MTCO2 (Cytochrome *c* oxidase subunit II) antibody	Abcam	Cat# ab110258, RRID:AB_10887758
COX4I1 (Anti-COX IV) antibody	Abcam	Cat# ab14744, RRID:AB_301443
COX5A antibody	Abcam	Cat# ab110262, RRID: AB_10861723
COX6B1 (Cytochrome *c* Oxidase subunit VIb) antibody	Proteintech	Cat# 11425-1-AP, RRID: AB_2085449
COX6C (Cytochrome *c* Oxidase subunit VIc) antibody	Abcam	Cat# ab110267, RRID:AB_10861117
ATP5F1B (ATPB) antibody	Abcam	Cat# ab14730, RRID:AB_301438
ATP5MC1 (Anti-ATP5G1/G2/G3 antibody) antibody	Abcam	Cat# ab180149
FLAG antibody	Sigma-Aldrich	Cat# F1804, RRID:AB_262044
HA Tag antibody	Thermo Fisher Scientific	Cat# 26183, RRID:AB_10978021
MRPL48 antibody	Proteintech	Cat# 14677-1-AP, RRID:AB_2282151
ATF4 (ATF4-human) antibody	Cell Signaling Technology	Cat# 11815, RRID:AB_2616025
eIF4EBP1 (4E-BP1 (53H11) Rabbit mAb) antibody	Cell Signaling Technology	Cat# 9644, RRID:AB_2097841
LC3B antibody	Abcam	Cat# ab48394, RRID:AB_881433
OPA-1 antibody	BD Biosciences	Cat# 612606, RRID:AB_399888
H3 (Histone H3) antibody	Cell Signaling Technology	Cat# 14269, RRID:AB_2756816
AMPKα (AMPK alpha (D5A2) Rabbit mAb) antibody	Cell Signaling Technology	Cat# 5831, RRID:AB_10622186
P- AMPKα T172 (AMPK-alpha, phospho (Thr172)) antibody	Cell Signaling Technology	Cat# 2535, RRID:AB_331250
S6K (p70 S6 Kinase) antibody	Cell Signaling Technology	Cat# 2708, RRID:AB_390722
P-S6K T389 (p70 S6 Kinase, phospho (Thr389)) antibody	Cell Signaling Technology	Cat# 9234, RRID:AB_2269803
S6 (Ribosomal Protein S6) antibody	Santa Cruz Biotechnology	Cat# sc-74576, RRID:AB_2181030
P-S6 S235/236 (Anti-p-Ribosomal Protein S6 Antibody (50.Ser 235/236)) antibody	Santa Cruz Biotechnology	Cat# sc-293144, RRID:AB_2895113
eIF2α (L57A5) antibody	Cell Signaling Technology	Cat# 2103, RRID:AB_836874
P-eIF2α S51 (Phospho-eIF2alpha (Ser51)) antibody	Cell Signaling Technology	Cat# 3398, RRID:AB_2096481
CS (Citrate synthetase) antibody	Abcam	Cat# ab129095, RRID:AB_11143209
Donkey anti-Mouse IgG (H + L) Highly Cross-Adsorbed Secondary Antibody, Alexa Fluor™ 680	Thermo Fisher Scientific	Cat# A10038, RRID:AB_2534014
Donkey anti-Rabbit IgG (H + L) Highly Cross-Adsorbed Secondary Antibody, Alexa Fluor™ 680	Thermo Fisher Scientific	Cat# A10043, RRID:AB_2534018
IRDye 800CW Donkey anti-Mouse IgG	LI-COR Biosciences	Cat# 926–32212, RRID:AB_621847
IRDye 800CW Donkey anti-Rabbit IgG	LI-COR Biosciences	Cat# 926–32213, RRID:AB_621848

**Chemicals, peptides, and recombinant proteins**		

Metafectene Pro	Biontex	Cat# T040–2.0
ISRIB	Merck	Cat# SML0843
Phosphatase Inhibitor Cocktail 2	Merck	Cat# P5726
ReadyShield® Protease and Phosphatase Inhibitor Cocktail	Merck	Cat# PPC2020
Benzonase® Nuclease	Merck	Cat# 70664
Immobilon®-FL PVDF membrane	Merck	Cat# 05317
EasyTag™ EXPRESS35S Protein Labeling Mix	PerkinElmer	Cat# NEG772002MC
Tetramethylrhodamine, Methyl Ester, Perchlorate (TMRM)	Thermo Fisher Scientific	Cat# T668
MitoTracker™ Deep Red FM	Thermo Fisher Scientific	Cat# M22426
FluoroBrite™ DMEM	Thermo Fisher Scientific	Cat# A1896701
CM-H2DCFDA (General Oxidative Stress Indicator)	Thermo Fisher Scientific	Cat# C6827
HBSS (10X), calcium, magnesium, no phenol red	Thermo Fisher Scientific	Cat# 14065056
DL-Dithiothreitol	Merck	Cat# D0632
Hydrogen peroxide solution	Merck	Cat# 216763

**Critical commercial assays**		

NAD+/NADH Glo assay	Promega	Cat# G9071

**Deposited data**		

Label-free quantification mass spectrometry data	Proteomics Identifications (PRIDE)	PXD046331

**Experimental models: Cell lines**		

Wild-type HEK293 cell line (wt)	ATCC	Cat#: CRL-1573, RRID:CVCL_0045
Wild-type HEK293 with stable AOX xeno-expression (wt + AOX)	this paper	N/A
*COX4I1/COX4I2* knock-out (KO) HEK293 cell line (4dKO)	Čunátová et al.^30^	N/A
4dKO with stable AOX xeno-expression (4dKO + AOX)	this paper	N/A
*COX6B1* KO HEK293 cell line (6BKO)	this paper	N/A
6BKO with stable AOX xeno-expression 6BKO + AOX	this paper	N/A
*ATP5F1B* KO HEK293 cell line (βKO)	Kovalčíkova et al.^50^	N/A
βKO with stable AOX xeno-expression (βKO + AOX)	this paper	N/A
*SDHA* KO HEK293 cell line (SDHA KO)	this paper	N/A
*SURF1* KO HEK293 cell line (SURF1 KO)	this paper	N/A
*ATP5F1D knock-down* HEK293 cell line (ATP5F1D KD)	Nuskova et al.^63^	N/A
*MLQ* KO HEK293 cell line (MLQ KO)	Tauchmannová et al.^64^	N/A
*USMG5* KO HEK293 cell line (DAPIT KO)	this paper	N/A
*TMEM70* KO HEK293 cell line (TMEM70 KO)	Kovalčíkova et al.^50^	N/A

**Oligonucleotides**		

siRNA targeting eIF4EBP1, Silencer Select	Thermo Fisher Scientific	assay IDs s4579, s223471, s223472
*COX6B1* gRNA sequence 1: AAGCGGCTGTCAAAAGGGGCGG	Merck	Cat# HSL0001377293
*COX6B1* gRNA sequence 2: GAACCAGACTAGAAACTGCTGG	Merck	Cat# HSR0001377298
*COX6B1* primer fw: TATTTGGGGACCAGGCTTGA	This study.	N/A
*COX6B1* primer rev*:* TGGACCTGAACAGCAGGTGA	This study.	N/A
*SDHA* gRNA sequence: TAAGGTGTGCAATAGCGAG	Merck	Cat# HSPD00000037631
*SDHA* primer fw: TGGGTGTTCTCGTGGTCTGT	This study.	N/A
*SDHA* primer rev*:* TCCTGTCACATCCACAGTCG	This study.	N/A
*SDHA* primer nested*:* CCACGCTGCTGTTCTCTGTT	This study.	N/A
*SURF1* gRNA sequence: ATGGTGGACCCTGTCCGGG	Merck	Cat# HSPD00000040751
*SURF1* primer fw: CTCCTTCACTTGTCTGGCCC	This study.	N/A
*SURF1* primer rev*:* GACAGTTCTCCAGAGGCAGG	This study.	N/A
*SURF1* primer nested*:* CTGGTGGGCTGTGTAGTCTG	This study.	N/A
*USMG5 sgRNA sequence 1: C∗A∗G∗UGUGUACUGGC* *CACAUA(TGTGTACTGGCCACATA)*	Synthego	Custom-made
*USMG5 sgRNA sequence 2: G∗A∗A∗AAAUCCUAACA* *UCAAAA(AAATCCTAACATCAAAA)*	Synthego	Custom/made
*USMG5* primer fw: GGAAAAATCCTAACATCAAAATGGC	This study.	N/A
*USMG5* primer rev*:* AGAATGAACTTAGCAGGGGCT	This study.	N/A

**Recombinant DNA**		

pcDNA™3.1 (+) Mammalian Expression Vector for AOX expression	Thermo Fisher Scientific	Cat# V79020
cDNA of AOX from A.nidulans	Perales-Clemente et al.^55^	N/A
eSpCas9-GFP system for *SDHA* and *SURF1* KO preparation	Merck	Cat# ESPCAS9GFP-1EA
CMV-CAS9D10A-2A-GFP Plasmid for *COX6B1* KO preparation	Merck	Cat# CAS9D10AGFPP
Mitochondria-targeted YFP	BD Biosciences	Cat# 6115-1
pLPCX mito roGFP2-Orp1	Addgene	RRID:Addgene_64992

**Software and algorithms**		

MaxQuant (v. 1.5.3.28) - MaxLFQ algorithm	Tyanova et al.,^76^ Cox et al.^77^	RRID:SCR_014485
Perseus (v. 2.0.9.0.)	Tyanova et al.^78^	RRID:SCR_015753
GraphPad Prism 8 software	GraphPad Software	RRID:SCR_002798
Image Lab software	Bio-Rad	RRID:SCR_014210
Gen5 software	BioTek	RRID:SCR_017317
FlowJo	BD Biosciences	RRID:SCR_008520
FIJI ImageJ	Schindelin et al.^79^	RRID:SCR_003070

### Resource availability

#### Lead contact

Further information and requests for resources and reagents should be directed to and will be fulfilled by the lead contact, Tomáš Mráček (tomas.mracek@fgu.cas.cz).

#### Materials availability

• This study did not generate new unique reagents.

#### Data and code availability

• Label-free quantification mass spectrometry (LFQ-MS) data have been deposited at PRIDE and are publicly available as of the date of publication. Accession numbers are listed in the key resources table.
• Original western blot images and microscopy data reported in this paper will be shared by the lead contact upon request.
• Any additional information required to reanalyze the data reported in this paper is available from the lead contact upon request.
• This paper does not report original code.

### Experimental model and study participant detail

HEK293 (ATCC CRL-1573) cells, a cell line with epithelial morphology isolated from the kidney of a human embryo, were maintained at 37°C and 5% CO_2_ atmosphere in DMEM/F-12 medium (Biowest, L0092) supplemented with 10% (v/v) FBS (Thermo Fisher Scientific, 10270-106), 40 mM HEPES, antibiotics (100 U/mL penicillin +100 μg/mL streptomycin, Thermo Fisher Scientific, 15140-122) and 50 μM uridine.

### Method details

#### Generation, culture, and treatment of HEK293 cellular models

COX4I1/COX4I2 (4dKO) and ATP5F1B (βKO) HEK293 (ATCC CRL-1573) knock-out (KO) cells were generated previously.^30^^,^^50^ COX6B1 KO (6BKO) cells were prepared using commercially available CAS9D10A system (CMV-CAS9D10A-2A-GFP Plasmid, Merck, USA). SDHA KO and SURF1 KO cells were generated using the commercially available eSpCas9-GFP system (ESPCAS9GFP-1EA). DAPIT KO model was prepared using Synthego Gene Knockout kit v2 according to manufactures instructions. For the details on the plasmids and guide RNAs, see the key resources table. Single-cell clones were obtained by fluorescence-activated cell sorting (FACS), in case of 6BKO, SDHA KO and SURF1 KO based on the medium fluorescence intensity of the GFP signal, and individual cell lines were then screened for the absence of the targeted protein using tricine - sodium dodecyl sulfate polyacrylamide gel electrophoresis (SDS-PAGE), western blotting (WB) and immunodetection. In the preselected clones, the presence of indel mutations in the CRISPR-Cas9 cleavage site resulting in the premature STOP codon was verified by Sanger sequencing (primers are at disposal in the key resources table). For alternative oxidase (AOX) expression in wt, 4dKO, 6BKO, and βKO cells (to produce wt AOX, 4dKO AOX, 6BKO AOX, βKO AOX), pcDNA3.1+ mammalian expression vector (Thermo Fisher Scientific, USA) containing the coding sequence of full-length AOX (from *Aspergillus nidulans),* followed by a C-terminal HA-tag,^55^ was transfected using Metafectene Pro (Biontex Laboratories GmbH). Stably transfected cells were selected with 1 mg/mL (4dKO, βKO) and 2 mg/mL (6BKO) G418. HEK293 cells were maintained at 37°C and 5% CO_2_ atmosphere in DMEM/F-12 medium (Biowest, L0092) supplemented with 10% (v/v) FBS (Thermo Fisher Scientific, 10270-106), 40 mM HEPES, antibiotics (100 U/mL penicillin +100 μg/mL streptomycin, Thermo Fisher Scientific, 15140-122) and 50 μM uridine. To inhibit mitochondrial ISR signaling, cells were treated with either ISRIB (1 μM, Merck, SML0843), or DMSO as a vehicle control, for at least 48 h. To silence the expression of *eIF4EBP1*, siRNA (Thermo Fisher Scientific, Silencer Select, assay IDs s4579, s223471,and s223472 mixed in equal quantities) was used according to.^50^

#### SDS-PAGE

Proteins separation under denaturing conditions was performed using tricine-sodium dodecyl sulfate polyacrylamide gel electrophoresis (SDS-PAGE). Protein samples were prepared from frozen cellular pellets as described.^48^^,^^80^ To examine the phosphorylation status of AMPKα, S6K, S6, and eIF2α proteins, cells were washed with ice-cold PBS and harvested in ice-cold RIPA buffer (150 mM NaCl, 1% Nonidet NP-40, 1% sodium deoxycholate, 0.1% SDS, 50 mM Tris, pH 8.0) supplemented with a phosphatase inhibitor cocktail (1:200, Sigma-Aldrich P5726), ReadyShield protease and phosphatase inhibitor cocktail (1:100, Sigma-Aldrich PPC2020) and benzonase nuclease (1:1000, Merck 70664). Samples were centrifuged (10,000 × g, 15 min), supernatant was mixed with the SLB buffer (sample lysis buffer; 2% (v/v) 2-mercaptoethanol, 4% (w/v) SDS, 50 mM Tris (pH 7.0), 10% (v/v) glycerol, 0.02% Coomassie Brilliant Blue R-250), and incubated at 65°C for 10 min 20–30 μg of protein were separated on 10% polyacrylamide gels (for separation of OPA-1 variants) or 12% polyacrylamide gels using the Mini-PROTEAN III apparatus (Bio-Rad, USA). Experiments were performed at least three times to assess the statistical significance of the results.

#### Native electrophoresis

For the separation of native protein complexes, blue native gel electrophoresis (BN-PAGE)^81^ was performed. Mitochondrial pellets were isolated from freshly harvested cells by hypotonic shock followed by differential centrifugation^82^ and solubilized using the mild detergent digitonin (6 g detergent/1 g protein) to preserve supercomplex association. Final samples (30 μg protein)^48^^,^^81^ were separated on a 4–13% polyacrylamide gradient gel using the Mini-PROTEAN III apparatus (Bio-Rad). Native electrophoresis analysis was performed in at least two independent experiments. For a limitation by an amount of βKO AOX cells, this model was not included in this experiment.

#### Western blotting (WB)

Proteins separated by SDS-PAGE or BN-PAGE were transferred on polyvinylidene difluoride (PVDF) membranes (Immobilon FL 0.45 μm, Merck) by semi-dry electroblotting (0.8 mA/cm^2^, 1 h) using a Transblot SD apparatus (Bio-Rad). Immunodetection was performed as described,^48^ using the primary and fluorescent secondary antibodies listed in the key resources table. The resulting signals were analyzed and quantified by Image Lab software (Bio-Rad).

#### Label-free quantification mass spectrometry analysis

Proteomic analysis was performed by the Proteomics Service Laboratory at the Institute of Physiology and the Institute of Molecular Genetics of the Czech Academy of Sciences. Label-free quantification mass spectrometry analysis (LFQ-MS) of cell pellets was performed in technical triplicates. Briefly, cellular pellets (100 μg of protein) were processed according to the protocol for in-solution trypsin digestion.^30^ About 1 μg of peptide digests were separated on a 50 cm C18 column using 2.5 h gradient elution and analyzed in a DDA mode on the Orbitrap Exploris 480 (Thermo Fisher Scientific) mass spectrometer. The resulting raw files were processed in MaxQuant (v. 1.5.3.28)^76^ with the label-free quantification (LFQ) algorithm MaxLFQ.^77^ LFQ-MS data are available via ProteomeXchange with identifier PXD046331.

#### Pulse-chase analysis

*In-vivo* metabolic pulse-chase labeling by ^35^S-methionine + ^35^S-cysteine using Express Protein Labeling Mix (PerkinElmer, USA) was used to study the synthesis and turnover of mtDNA-encoded proteins. Analysis of wt, 4dKO, 6BKO, βKO, 4dKO AOX, and 6BKO AOX cells was performed in three independent experiments. Cells were labeled for 3 h in methionine- and cysteine-free DMEM with anisomycin (100 μg/mL) and the radioactive labeling mix (100 μCi/mL). After, non-labeled methionine and cysteine (final concentration 250 μM) were added, and the cells were incubated for 15 min. The cells were washed with non-labeled methionine and cysteine (both 250 μM) in PBS, and “pulse” samples were harvested. Parallel dishes with labeled cells were incubated for 24 h in a complete non-labeling DMEM medium and then harvested as “chase” samples. SDS samples were prepared from cell pellets and were separated (70 μg protein) using a 16% polyacrylamide gel in a large format Hoefer SE 600 Chroma Vertical Electrophoresis System (Thermo Fisher Scientific), followed by WB. Radioactivity was detected by exposing the PVDF membrane to a Storage Phosphor Screen BAS-IP SR 2025 E for 14 days (GE Healthcare), which was then scanned using an Amersham Typhoon biomolecular imager (GE Healthcare, USA). Signals were quantified by Image Lab software (Bio-Rad) and normalized to the immunodetection signal of a specific antibody against CS. For a limitation by an amount of βKO AOX cells, this model was not included in this experiment.

#### Evaluation of metabolic fluxes

Seahorse Extracellular Flux (XF) Analyzer (Agilent Technologies, USA) was used to assess intact mitochondrial respiration and glycolytic rate in parallel, as described.^30^^,^^48^ Briefly, cells (2–3.5 × 10^4^) were seeded in pentaplicates on poly-L-lysine-coated wells of a 24-well measuring plate and incubated overnight under standard conditions. Before the evaluation, wells were washed with 1 mL of DMEM (Merck, cn: D5030, pH 7.4, 37°C) supplemented with 0.2% (w/w) BSA, 500 μL of the same medium was added and incubated for 30 min at 37°C. Basal metabolic rate and rates after subsequent additions of the following substrates and inhibitors were recorded: glucose (10 mM), oligomycin (1 μM), FCCP (1 μM), and a mixture of rotenone (1 μM), antimycin A (1 μg/mL), 2-deoxyglucose (100 mM) and Hoechst 33342 (5 μg/mL). Images of whole wells were captured using a Cytation 3 Cell Imaging Reader (BioTek, USA) and analyzed using the Gen5 software (BioTek). The oxygen consumption (OCR) and extracellular acidification (ECAR) rates were normalized to the cell count. In the case of 4dKO ± AOX and βKO ± AOX, 2-deoxyglucose addition led to cell detaching that prevented cell count. Sample evaluations were performed in at least three independent experiments.

#### Evaluation of mitochondrial membrane potential

Mitochondrial membrane potential (Δψ_m_) was analyzed in intact cells using slow redistribution dye tetramethylrhodamine methyl ester (TMRM, cn: T668, ThermoFisher). Aliquots of cells (0.15 mg) were diluted in FluoroBrite DMEM media (cn: A1896701, ThermoFisher) supplemented with 1 mM sodium pyruvate, 2 mM glutamine, 10 nm TMRM and 50 nM MitoTracker Deep Red FM (MTDR, cn: M22426, ThermoFisher). Where indicated, an uncoupler carbonyl cyanide 4-(trifluoromethoxy) phenylhydrazone (FCCP, cn: C2920, Merck) was added. The aliquotes were incubated for 30 min at 37°C under constant mixing and fluorescence intensities were measured by BD LSRII flow cytometer (BD Biosciences). Excitation laser wavelengths were 561 nm (TMRM) and 633 nm (MTDR) and fluorescence intensity was detected using band pass emission filters 610/20 nm (TMRM) and 660/20 (MTDR). The signal of Δψ_m_-dependent TMRM was normalized to the Δψ_m_-independent signal of MTDR by FlowJo software (v 10.9.0, BD Biosciences). The columns represent the mean of TMRM/MTDR ratio from three independent measurements, in each measurement 10 000 cells were analyzed. For a limitation by an amount of βKO AOX cells, this model was not included in this experiment.

#### Assessment of NAD^+^/NADH ratio

The ratio of oxidized and reduced form of nicotinamide adenine dinucleotide (NAD^+^/NADH) was assessed using the NAD^+^/NADH Glo assay (Promega, Madison, WI, USA) according to the manufacturer’s protocol. The assays were performed in at least three independent experiments.

#### Confocal microscopy to study mitochondrial redox state

Cells were seeded on poly-L-lysine coated 25 mm glass coverslips at a density of 250 000 cells per glass. After overnight incubation, they were transfected (DNA: metafectene, 1 μg: 4 μl) with mitochondria-targeted roGFP (Addgene # 64992), a ratio-metric sensor that allows to monitor mitochondrial redox state.^83^ 1–2 days after transfection, the coverslips were mounted into measuring chamber and experimental media was added (HBSS media [Thermo Fisher Scientific, 14065-049] supplemented with 20 mM HEPES and 2 mM glutamine). The cells were imaged at 37°C using Leica SP8 WLL MP laser scanning confocal microscope HC PL APO 63× water immersion objective (1.20 N.A.)) with the pinhole set to 5 airy units. The imaging was performed according to^84^ with slight modifications. The fluorescence of reduced (Exc/Em: 405/520 nm) or oxidized (Exc/Em: 490/520 nm) state of the sensor was recorded, pictures were acquired approximately every 30 s. The baseline fluorescence was measured for 4 min followed by the addition of dithiothreitol (DTT, final concentration 1 mM; Merck, D0632) to reach the maximal reduction of roGFP. After 15 min, the hydrogen peroxide (H_2_O_2_, final concentration 2 mM; Merck, 216763) was added and the maximal roGFP oxidation was recorded for 15 min. Each cell line was analyzed in at least three independent experiments, minimum of 11 cells per experiment were recorded. The videos were processed with Fiji^79^ – after the threshold setting, the individual cells were selected and the mean intensities from oxidized and reduced states were evaluated. The intensity ratio of both states (oxidized/reduced) was calculated in Excel and the basal values were expressed as percentage of the range (maximal oxidation – maximal reduction). For a limitation by an amount of βKO AOX cells, this model was not included in this experiment.

#### Evaluation of mitochondrial ROS production

Generation of mitochondrial ROS was assessed by monitoring 1 μM CM-H_2_DCFDA (chloromethyl derivative of 2′,7′-dichlorodihydrofluorescein diacetate, Thermo Fisher Scientific) fluorescence.^48^ Cells were seeded on poly-L-lysine-coated 24-well plates (3 × 10^5^). On the second day, cells were incubated in parallel with either CM-H_2_DCFDA (“basal”) or CM-H_2_DCFDA and FCCP (“FCCP”, 1 μM) for 2 h at 37°C. An increase in fluorescence was recorded using an Infinite M200 plate reader (Tecan Group Ltd., Switzerland) at 495/525 nm, and the difference between “basal” and “FCCP” CM-H_2_DCFDA fluorescence was calculated and attributed to mitochondrial ROS production.^85^ At least three independent replicates were measured for ROS evaluation.

#### Determination of ATP, ADP, and AMP levels

The levels of adenine nucleotides in cellular extracts were quantified by HPLC assay. Briefly, cells grown to confluency on a 100 mm Petri dish were flesh frozen by liquid nitrogen. Subsequently, cells were extracted with 1.5 mL of 6% (v/v) perchloric acid. The extract was centrifuged for 10 min at 10,000 *g* to remove cell debris. Supernatants were neutralized to pH 7 by 0.4 M triethanolamine/1.8 M KOH, centrifuged again to remove the perchlorate precipitate, and stored at –80°C. Separation and quantification of ATP, ADP, and AMP were performed as described.^86^

#### Transmission electron microscopy to visualize mitochondrial cristae ultrastructure

The cells were seeded in 12-well plates, allowed to reach 70–80% confluency, fixed with 2.5% glutaraldehyde in 0.1M sodium cacodylate pH 7.4, treated with 1% OsO4 and 1% K_4_Fe(CN)_6_ in 0.1 M sodium cacodylate, stained with 0.25% uranyl acetate overnight, dehydrated in ethanol, and embedded in Durcupan Epon. Samples were sectioned by Ultracut microtome and visualized using the JEOL JEM-1200 EX transmission electron microscope. Images were analyzed by FIJI ImageJ.^79^ At least 40 mitochondria were analyzed per cell line with the range of 150–280 individual cristae.

#### Confocal microscopy to study mitochondrial reticulum organization

Cells were seeded on glass coverslips in 6-well plates and transfected the following day with mitochondria-targeted YFP (BD Biosciences, 6115-1). After overnight incubation, cells were imaged at 37°C in FluoroBrite DMEM medium (Thermo Fisher Scientific, A1896701) supplemented with 2 mM glutamine using a Leica SP8 WLL MP laser scanning confocal microscope with an HC PL APO 63× water immersion objective (1.20 N.A.). The collected z-stacks were processed and analyzed with the 3-D features of the ImageJ/Fiji plug-in “Mitochondria Analyzer”,^87^ using a C-value of 4 and a block size of 1.3.

### Quantification and statistical analysis

Data were analyzed and visualized in GraphPad Prism 8 software (GraphPad Software). Downstream analysis of LFQ-MS data was performed in Perseus (v. 2.0.9.0.)^78^ and data were further visualized in GraphPad Prism 8. One sample t-test (Hash represents *p*-value: # <0.05) or One-way ANOVA (Asterisks represent *p*-value: ∗ <0.05; ∗∗ <0.01; ∗∗∗ <0.001) were used, as applicable in individual figures description. Significant changes between individual samples and wt are represented by a symbol over the corresponding bar, while comparison between KO models without and with AOX expression is indicated by a star symbol with a connecting line. Data shown in the graphs represent the mean values ±SD, with an exception of evaluation of metabolic fluxes, which are represented as mean ± SEM, of at least 3 independent experiments.
