# Supplementary material for: Mitochondrial translation is the primary determinant of secondary mitochondrial complex I deficiencies
Source: iScience. 2024 Jul 19;27(8):110560. doi: 10.1016/j.isci.2024.110560 (PMC11342289; doi:10.1016/j.isci.2024.110560)
Supplement: Document S1. Figures S1–S6 [file mmc1.pdf]

iScience, Volume *27*

Supplemental information

Mitochondrial translation is the primary determinant of secondary mitochondrial complex I deficiencies

Kristýna Čunátová, Marek Vrbacký, Guillermo Puertas-Frias, Lukáš Alán, Marie Vanišová, María José Saucedo-Rodríguez, Josef Houštěk, Erika Fernández-Vizarra, Jiří Neužil, Alena Pecinová, Petr Pecina, and Tomáš Mráček

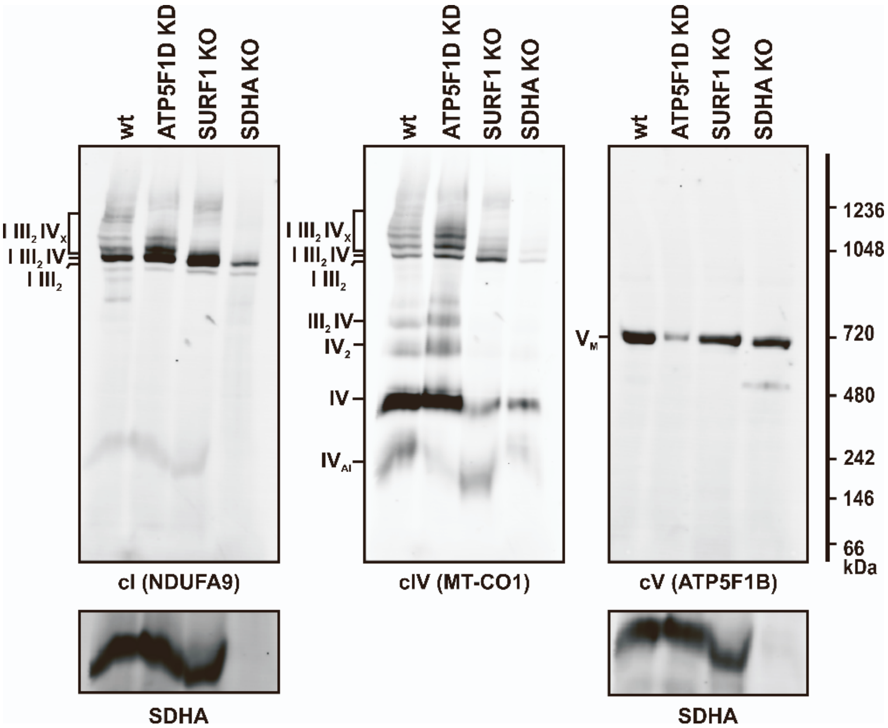

Figure S1. Secondary cI decline is not associated with mild OXPHOS deficiencies but occurs in cII-lacking cells.

Blue-native (BN)-PAGE/WB detection of OXPHOS complexes: cI (NDUFA9 antibody), cIV (MT-CO1 antibody), and cV (ATP5F1B antibody). Antibody against cII (SDHA) was used as a loading control. MT-CO1 antibody was developed on the same membrane as NDUFA9 after removal (stripping) of NDUFA9 and SDHA primary antibodies and has the same (SDHA) loading control. wt, SDHA KO (cII), SURF1 KO (cIV) and ATP5F1D KD (cV) cells were utilized.

Related to Figure 1

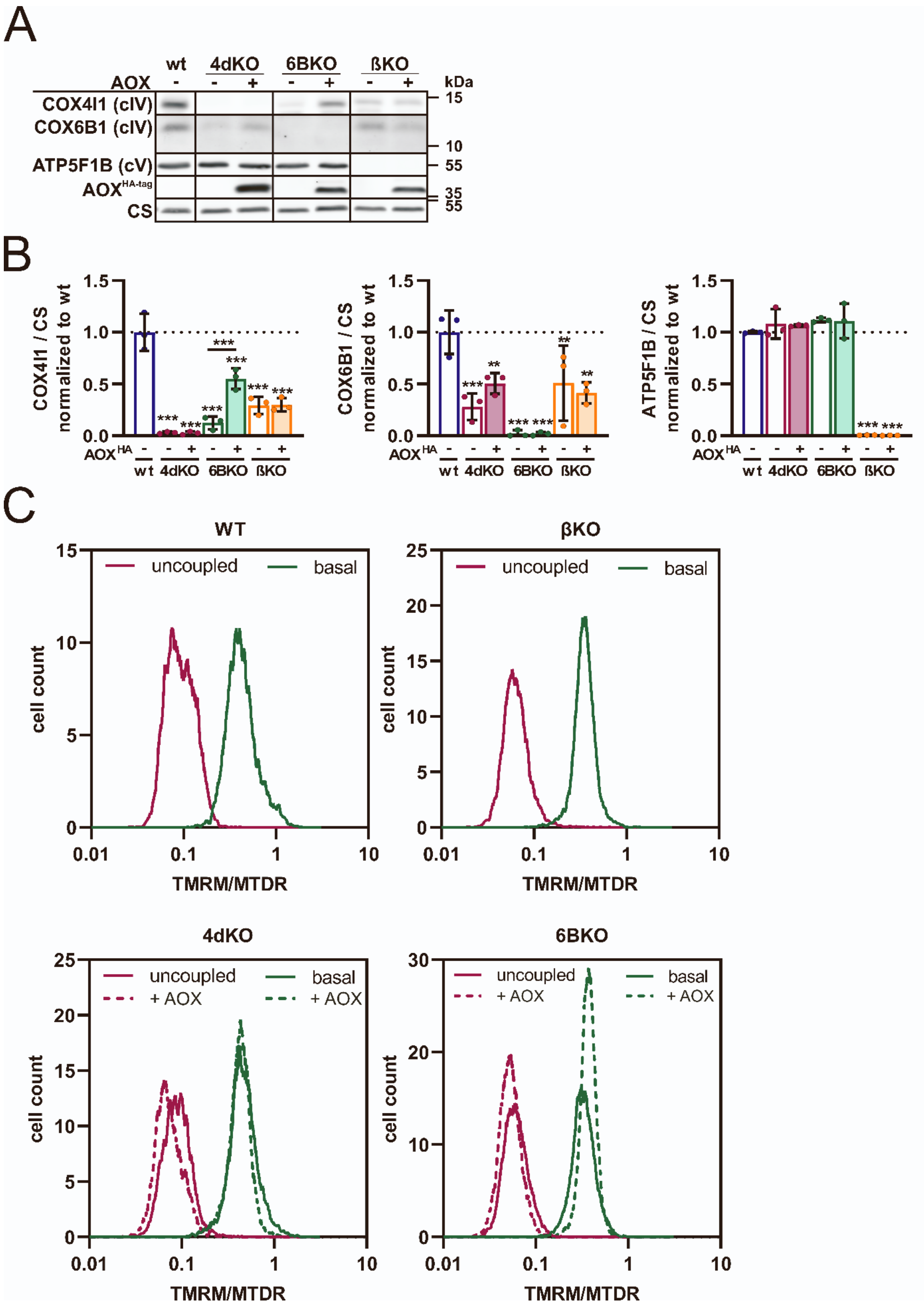
A
wt
4dKO
6BKO
ßKO
AOX
kDa
COX4I1 (cIV)
COX6B1 (cIV)
ATP5F1B (cV)
AOX^HA-tag
CS
15
10
55
35
55
B
COX4I1 / CS normalized to wt
COX6B1 / CS normalized to wt
ATP5F1B / CS normalized to wt
AOX^HA
C
WT
βKO
4dKO
6BKO
uncoupled
basal
+ AOX
cell count
TMRM/MTDR

Figure S2. Validation of the cellular models used in the present study.

(A) Representative SDS-PAGE/WB analysis of the protein steady-state levels of cIV subunits (COX4I1, COX6B1), cV subunit ATP5F1B and HA-tagged AOX (AOXHA-tag) in whole cell-lysates. Citrate synthase (CS) antibody was used as a loading control.
(B) Quantification of COX4I1, COX6B1, and ATP5F1B signals from SDS-PAGE/WB analysis normalized to CS.
(C) Mitochondrial membrane potential in basal (green) and uncoupled (magenta) states in wt, βKO, 4dKO, 6BKO cells (full line), and 4dKO AOX and 6BKO AOX cells (dashed line).

Related to Figure 2

One-way ANOVA ($p < 0.05$; ** $p < 0.01$; *** $p < 0.001$) was performed ($n = 3$). Data are represented as mean ± SD. wt, 4dKO, 6BKO (cIV) and βKO (cV) ± AOX cell lines were utilized.*

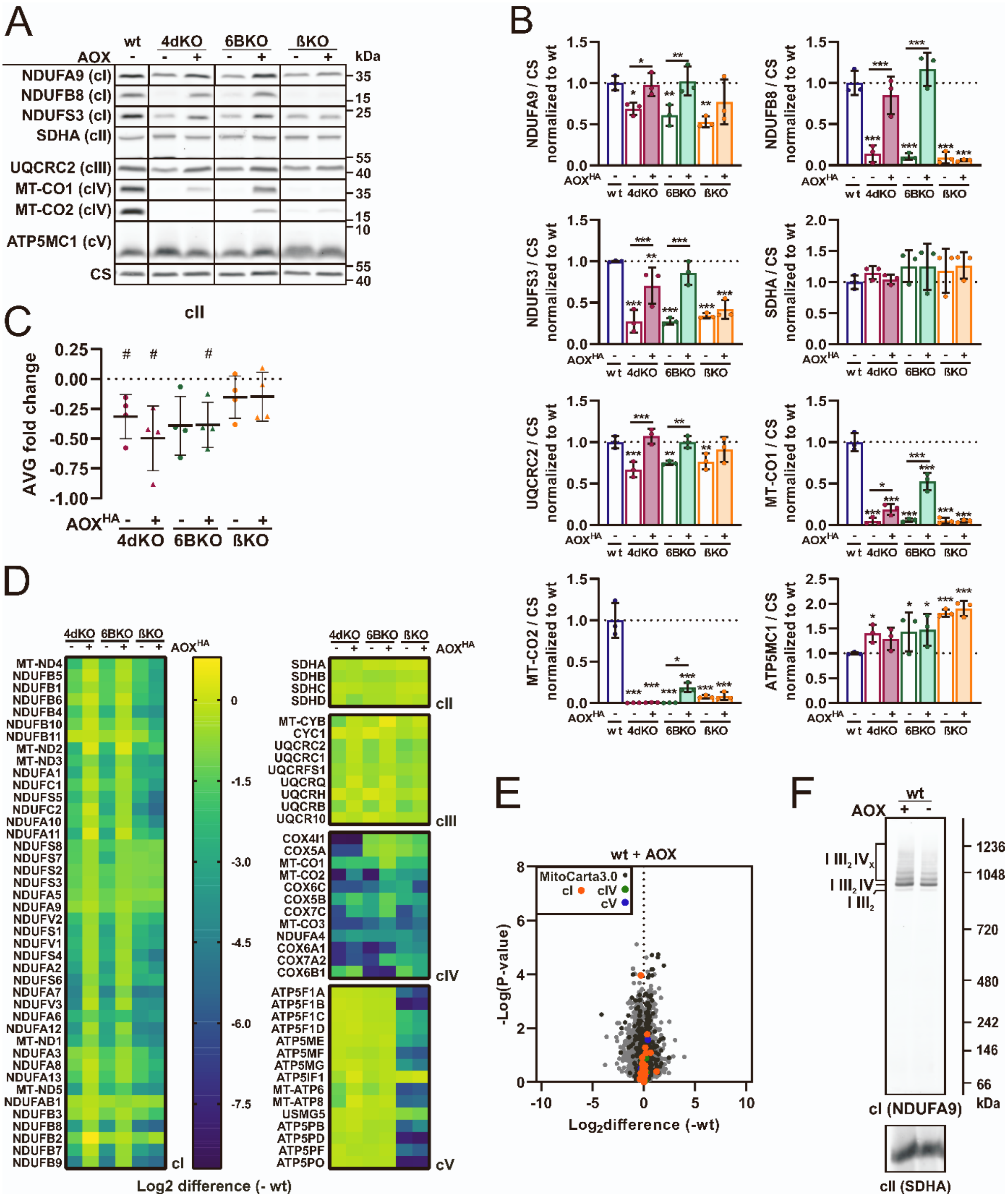
A
wt
4dKO
6BKO
ßKO
AOX
kDa
NDUFA9 (cI)
NDUFB8 (cI)
NDUFS3 (cI)
SDHA (cII)
UQCRC2 (cIII)
MT-CO1 (cIV)
MT-CO2 (cIV)
ATP5MC1 (cV)
CS
B
NDUFA9 / CS normalized to wt
NDUFB8 / CS normalized to wt
NDUFS3 / CS normalized to wt
SDHA / CS normalized to wt
UQCRC2 / CS normalized to wt
MT-CO1 / CS normalized to wt
MT-CO2 / CS normalized to wt
ATP5MC1 / CS normalized to wt
AOXHA
C
cII
AVG fold change
D
Log2 difference (- wt)
cI
cII
cIII
cIV
cV
E
wt + AOX
MitoCarta3.0
cI
cIV
cV
-Log(P-value)
Log2difference (-wt)
F
cI (NDUFA9)
cII (SDHA)

Figure S3. AOX expression restores cI levels in cIV-deficient cells.

(A) Representative SDS-PAGE/WB analysis of the protein steady-state levels of OXPHOS complex subunits in whole cell-lysates. CS antibody was used as a loading control.
(B) Quantification of OXPHOS complexes subunits signals from SDS-PAGE/WB analysis normalized to CS.
(C) LFQ-MS based average fold-change of subunits of cII.
(D) LFQ-MS based average fold-change of subunits forming OXPHOS complexes.
(E) LFQ-MS analysis of differential protein content between wt and wt + AOX cells. All analyzed proteins (grey), MitoCarta3.0 annotated proteins (black), and subunits of cI (orange), cIV (green) and cV (blue).
(F) BN-PAGE/WB detection of OXPHOS complexes cI (NDUFA9 antibody) and cII (SDHA, used as a loading control) (n = 3).

Related to Figure 3

One sample t-test (# $p < 0.05$), or One-way ANOVA ($p < 0.05$; ** $p < 0.01$; *** $p < 0.001$) was performed (n = 3). Data are represented as mean ± SD. wt, 4dKO, 6BKO (cIV) and βKO (cV) ± AOX cell lines were utilized.*

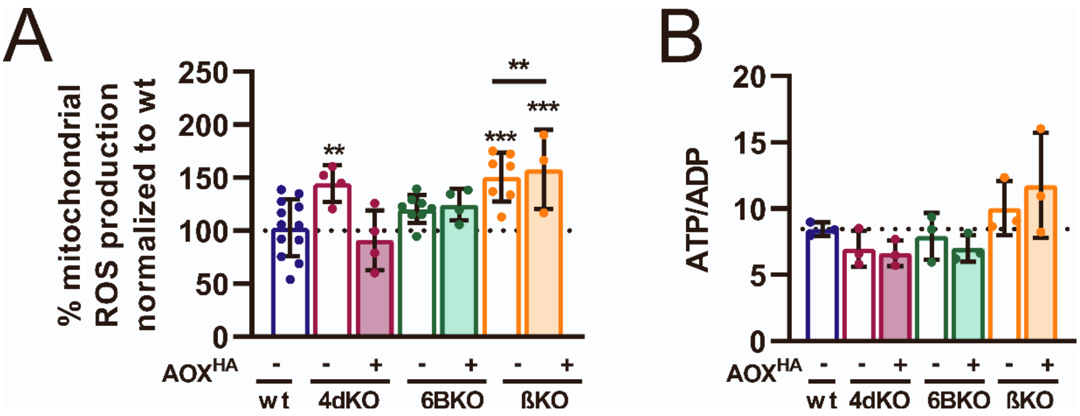

Figure S4. Energetic status of the studied cellular models is comparable to the wt.

(A) Mitochondrial ROS production normalized to wt ($n \geq 3$).
(B) ATP to ADP ratio of studied cellular models ($n = 3$).

Related to Figure 4

One-way ANOVA ($p < 0.05$; ** $p < 0.01$; *** $p < 0.001$) was performed ($n = 3$). Data are represented as mean ± SD. wt, 4dKO, 6BKO (cIV) and βKO (cV) ± AOX cell lines were utilized.*

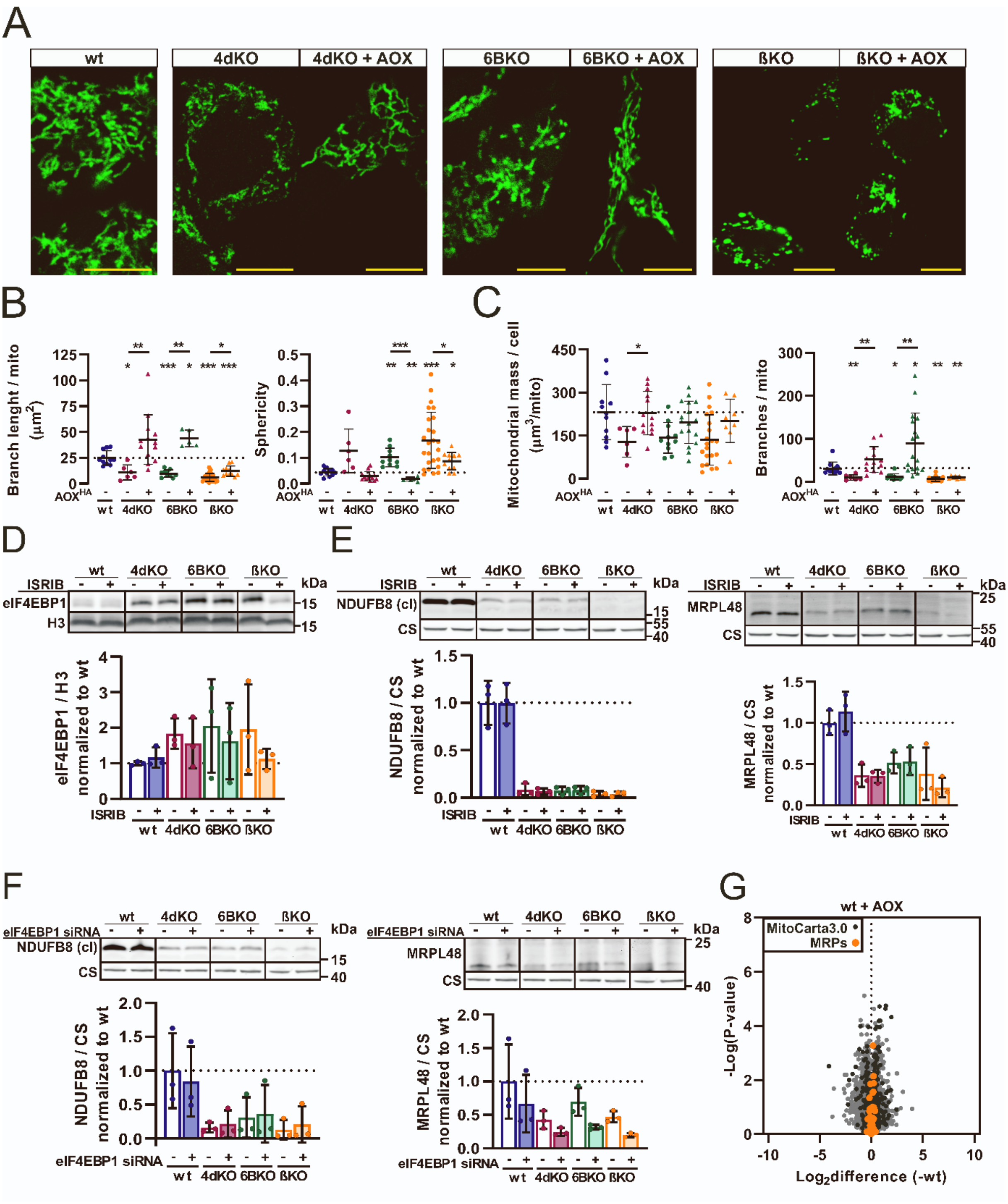
A
wt
4dKO
4dKO + AOX
6BKO
6BKO + AOX
ßKO
ßKO + AOX
B
Branch lenght / mito (µm2)
Sphericity
AOXHA
wt 4dKO 6BKO ßKO
C
Mitochondrial mass / cell (µm3/mito)
Branches / mito
D
ISRIB
eIF4EBP1
H3
kDa
eIF4EBP1 / H3 normalized to wt
E
NDUFB8 (cl)
CS
NDUFB8 / CS normalized to wt
MRPL48
MRPL48 / CS normalized to wt
F
eIF4EBP1 siRNA
G
wt + AOX
MitoCarta3.0
MRPs
-Log(P-value)
Log2difference (-wt)

Figure S5. Mitochondrial ISR is triggered in OXPHOS-deficient cells, but is responsible for neither cI, nor MRPs decrease.

(A) Representative images of mitochondria obtained by confocal microscopy.
(B) Confocal microscopy-based analysis of mitochondrial morphology, precisely Branch length per mitochondria and Sphericity ($n \geq 6$).
(C) Mitochondrial mass per cell and Branches per mitochondria based on confocal microscopy analysis ($n \geq 6$).
(D) Representative SDS-PAGE/WB analysis of the protein steady-state level of eIF4EBP1 in whole cell-lysates of the cells with DMSO addition (-), or after 48h incubation with 1uM ISRIB (+). The graph represents the quantification of the eIF4EBP1 signal normalized to H3 (n = 3).
(E) Representative SDS-PAGE/WB analysis of the protein steady-state level of NDUFB8 and MRPL48 in whole cell-lysates of the cells with DMSO addition (-), or after 48h incubation with 1uM ISRIB (+). The graphs represent the quantification of NDUFB8 and MRPL48 signals normalized to CS (n = 3).
(F) Representative SDS-PAGE/WB analysis of the protein steady-state level of NDUFB8 and MRPL48 in whole cell-lysates of the cells transfected with scrambled siRNA (-), or transfected with eIF4EBP1 siRNA (+). The graphs represent quantification of NDUFB8 and MRPL48 signals normalized to CS (n = 3).
(G) LFQ-MS analysis of differential protein content between wt and wt + AOX cells. All analyzed proteins (grey), MitoCarta3.0 annotated proteins (black), and MRP subunits (orange).

Related to Figure 5 (panels A-F) and Figure 6 (panel G)

One-way ANOVA ($p < 0.05$; ** $p < 0.01$; *** $p < 0.001$) was performed. Data are represented as mean ± SD. wt, 4dKO, 6BKO (cIV) and βKO (cV) ± AOX cell lines were utilized.*

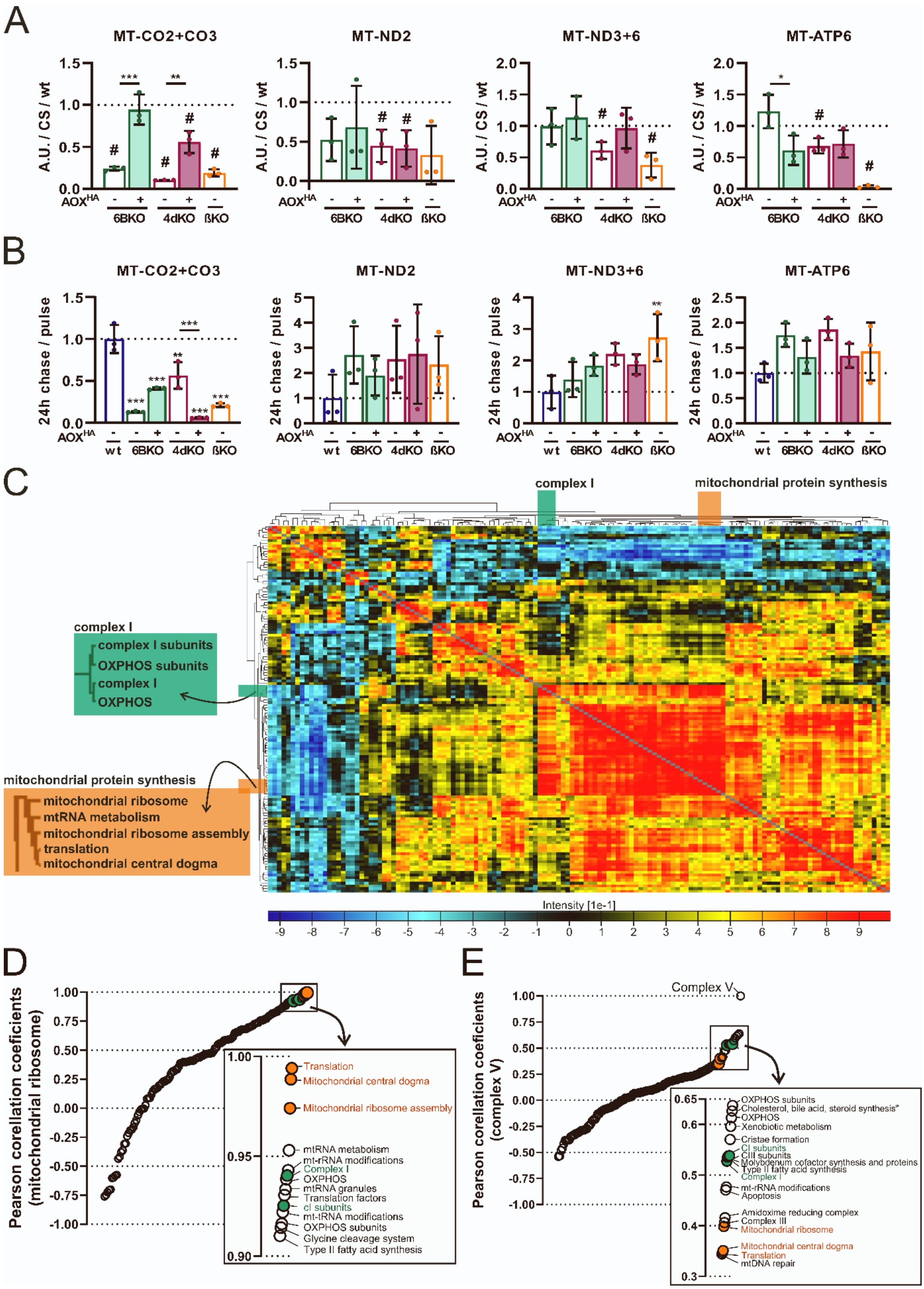
A
MT-CO2+CO3
MT-ND2
MT-ND3+6
MT-ATP6
A.U. / CS / wt
AOX^HA
6BKO
4dKO
ßKO
B
24h chase / pulse
wt
C
complex I
mitochondrial protein synthesis
complex I subunits
OXPHOS subunits
complex I
OXPHOS
mitochondrial ribosome
mtRNA metabolism
mitochondrial ribosome assembly
translation
mitochondrial central dogma
Intensity [1e-1]
D
Pearson corellation coeficients (mitochondrial ribosome)
Translation
Mitochondrial central dogma
Mitochondrial ribosome assembly
mtRNA metabolism
mt-rRNA modifications
Complex I
OXPHOS
mtRNA granules
Translation factors
cI subunits
mt-tRNA modifications
OXPHOS subunits
Glycine cleavage system
Type II fatty acid synthesis
E
Pearson corellation coeficients (complex V)
Complex V
OXPHOS subunits
Cholesterol, bile acid, steroid synthesis"
OXPHOS
Xenobiotic metabolism
Cristae formation
CI subunits
CIII subunits
Molybdenum cofactor synthesis and proteins
Type II fatty acid synthesis
Complex I
mt-rRNA modifications
Apoptosis
Amidoxime reducing complex
Complex III
Mitochondrial ribosome
Mitochondrial central dogma
Translation
mtDNA repair

Figure S6. Secondary decline of cI is due to mitochondrial protein synthesis attenuation, rather than its degradation.

(A) Quantification of pulse ^{35}S *in vivo* labeling signal of representative cIV (MT-CO2+CO3), cI (MT-ND2, MT-ND3+6), and cV (MT-ATP6) subunits normalized to CS.
(B) Quantification of 24h chase to pulse ratio of ^{35}S *in vivo* labeling signal of representative cIV (MT-CO2+CO3), cI (MT-ND2, MT-ND3+6), and cV (MT-ATP6) subunits.
(C) Heatmap of Pearson correlation coefficients of LFQ-MS based average fold-changes of MitoCarta3.0 annotated categories relative to wt in HEK293-cell line based models of i) sever deficiency: SDHA KO (cII, n = 3), 4dKO (cIV, n = 3), 6BKO (cIV, n = 3), βKO (cV, n = 3), and ii) mild deficiency: SURF1 KO (cIV, n = 3), ATP5F1D KD (cV, n = 2), MLQ KO (cV, n = 2), DAPIT KO (cV, n = 3), TMEM70 KO (cV, n = 3).
(D) Pearson correlation coefficients of LFQ-MS based average fold-changes comparison of mitochondrial ribosome with MitoCarta3.0 annotated categories relative to wt in HEK293-cell line based models of i) sever deficiency: SDHA KO (cII, n = 3), 4dKO (cIV, n = 3), 6BKO (cIV, n = 3), βKO (cV, n = 3), and ii) mild deficiency: SURF1 KO (cIV, n = 3), ATP5F1D KD (cV, n = 2), MLQ KO (cV, n = 2), DAPIT KO (cV, n = 3), TMEM70 KO (cV, n = 3).
(E) Pearson correlation coefficients of LFQ-MS based average fold-changes comparison of complex V with MitoCarta3.0 annotated categories relative to wt in HEK293-cell line based models of i) sever deficiency: SDHA KO (cII, n = 3), 4dKO (cIV, n = 3), 6BKO (cIV, n = 3), βKO (cV, n = 3), and ii) mild deficiency: SURF1 KO (cIV, n = 3), ATP5F1D KD (cV, n = 2), MLQ KO (cV, n = 2), DAPIT KO (cV, n = 3), TMEM70 KO (cV, n = 3).

Related to Figure 7

One sample t-test (# $p < 0.05$), or One-way ANOVA ($p < 0.05$; ** $p < 0.01$; *** $p < 0.001$) was performed (n = 3). Data are represented as mean ± SD. wt, 4dKO, 6BKO (cIV) and βKO (cV) ± AOX cell lines were utilized.*
